# Exploring chaos and sensitivity in the Ivancevic option pricing model through perturbation analysis

**DOI:** 10.1371/journal.pone.0312805

**Published:** 2024-11-26

**Authors:** Adil Jhangeer, Ali R. Ansari, Ariana Abdul Rahimzai, Beenish, Abdul Qadeer Khan

**Affiliations:** 1 IT4Innovations, VSB – Technical University of Ostrava, Ostrava, Poruba, Czech Republic; 2 Centre for Applied Mathematics and Bioinformatics (CAMB), Gulf University for Science and Technology, Mubarak Al-Abdullah, Kuwait; 3 Department of Mathematics, Education Faculty, Laghman University, Mehtarlam City, Laghman, Afghanistan; 4 Department of Mathematics, Quaid-I-Azam University, Islamabad, Pakistan; 5 Department of Mathematics, University of Azad Jammu and Kashmir, Muzaffarabad, Pakistan; Federal University of Technology - Parana, BRAZIL

## Abstract

This study explores the Ivancevic Option Pricing Model, a nonlinear wave-based alternative to the Black-Scholes model, using adaptive nonlinear Schrödingerr equations to describe the option-pricing wave function influenced by stock price and time. Our focus is on a comprehensive analysis of this equation from multiple perspectives, including the study of soliton dynamics, chaotic patterns, wave structures, Poincaré maps, bifurcation diagrams, multistability, Lyapunov exponents, and an in-depth evaluation of the model’s sensitivity. To begin, a wave transformation is applied to convert the partial differential equation into an ordinary differential equation, from which soliton solutions are derived using the $\left(\frac{G^{\prime }}{G}\right)$ method. We explore various forms of the option price function at different time points, including singular-kink, periodic, hyperbolic, trigonometric, exponential, and complex solutions. Furthermore, we simulate 3D surface plots and 2D graphs for the real, imaginary, and modulus components of some of the obtained solutions, assigning specific parameter values to enhance visualization. These graphical representations offer valuable insights into the dynamics and patterns of the solutions, providing a clearer understanding of the model’s behavior and potential applications. Additionally, we analyze the system’s dynamic behavior when a perturbing force is introduced, identifying chaotic patterns using the Lyapunov exponent, Sensitivity, multistability analysis, RK4 method, wave structures, bifurcation diagrams, and Poincaré maps.

## Introduction

The potential of nonlinear models for capturing the dynamics of many different scientific and natural phenomena, including surface waves from the sea, gravity waves, acoustic electricity waves, shallow water motion, fluid mechanics, and nonlinear optics, has attracted a great deal of emphasis [1–3]. Nonlinear partial differential equations (NLPDEs) are crucial for modeling these phenomena and solving many real-world challenges. The pursuit of analytic solutions to NLPDEs has become a well-studied, continuously advancing, and complex area of research, with several notable contributions [4, 5]. In recent years, several authors have used some approaches to work on the solution of several NPDEs. Some among these include the generalized tanh–coth method [6–8], the extended hyperbolic function technique [9], the new auxiliary equation method [10], the new extended direct algebraic technique [11], among other [12–17].

In mathematical finance, Black-Scholes is the conventional model that details the temporal dynamics of financial equity, for instance, stock options [18]. It prescribes that the asset price *R* = *R*(*τ*), is governed by the geometric Brownian motion model with constant parameters *σ*_1_ and *σ*_2_, and embodies the efficient market hypothesis that there are no risks-less profits possible and that markets are free from trading costs [19, 20]:

$$\begin{array}{c} dR=R\left(\sigma_{1}d\tau +\sigma_{2}dW_{\tau }\right),\;\;R\in \;\left[0,\infty \right),\;\sigma_{1},\sigma_{2}\;>\;0,\;\;0<\tau <T^{⋆}. \end{array}$$ (1)

*W*_*τ*_ represents the mean rate of exchange of *R*, the level of volatility, and a conventional Brownian motion, correspondingly. Suppose *U*^⋆^ = *U*^⋆^(*R*, *τ*), the Black-Scholes PDE (1) can be expressed as [21]:

$$\begin{array}{c} \left\{ \begin{array}{c} \begin{array}{cc} & U_{\tau }^{⋆}+r_{1}RU_{R}^{⋆}+0.5R^{2}\sigma_{2}^{2}U_{RR}^{⋆}-r_{1}U^{⋆}=0,\\ & U^{⋆}\left(0,\tau \right)=0,\;\;U^{⋆}\;\to \;0\;\;as\;\;R\;\to \;\infty ,\\ & U^{⋆}\left(R,\tau \right)=Max\left(R-F,0\right)\;\;\;F\in R,\\ & R\left(\tau \right)=R_{0}e^{U^{⋆}-\frac{\sigma_{2}^{2}}{2}}\tau +\sigma_{2}W_{\tau },\;\;R_{0}=R\left(0\right). \end{array} \end{array}\right. \end{array}$$ (2)

Research scholars have stressed Eq (2) and have investigated its solutions through various techniques [22–24]. Vukovic [25] took quantum connection just a couple of weeks ago, linking Schrödinger and Black-Scholes equations where the former’s Hamiltonian returns complex eigenvalues while Black-Scholes Hamiltonian does so. Furthermore, it has been demonstrated that the Black-Scholes equation can be derived by transforming financial market equations into the Schrödinger equation from quantum mechanics. This goes further to underscore the fact that while the Schrödinger equation demands a complex state function, Black-Scholes requires a real-valued state of options.

In 1900, Louis Bachelier himself proposed an option pricing model where the price of an underlying asset can only undergo stochastic movements along a curve without the presence of any deterministic trend. It has been generalized by many researchers, as mentioned in [26], mainly to depict the relation of the option price with the price of the underlying asset, time to expiration, volatility, and risk-free interest rate. Ivancevic [27] used the quantum-probability formation to address the time-dependent Schrödinger equation, coming up with a wave-form non-linear model after its adaptive-dependent model. This option pricing model is known as the Ivancevic option pricing model (IOPM) and offers the equal probability density function of the stock option’s market value [28, 29]:

$$\begin{array}{c} i\Theta_{\tau }+\frac{\sigma_{2}\Theta_{SS}}{2}+\beta_{0}{|\Theta |}^{2}\Theta =0,\;\;i=\sqrt{-1}. \end{array}$$ (3)

In this context, Θ = Θ(*S*, *τ*) is the option price function at time *τ*. While at |Θ| = |Θ(*S*, *τ*)| means the probability density for the option price regarding the stock price and time. The symbol *σ*_2_ denotes a constant or stochastic process for the volatility coefficient. The coefficient *β*_0_ is called the Landau coefficient, which reflects adaptability to the market. The model (3) becomes linear when *β*_0_ = 0. Moreover, Eq (3) relates the economy and option pricing. Some solutions to Eq (3) are analyzed in [30] by using the trial function method, the tanh expansion method, and the direct perturbation method. Various research methodologies have been utilized to investigate the Ivancevic option pricing model. In [31], the authors explored soliton solutions using the modified exponential function method. Similarly, the unified auxiliary equation Method was applied in [32] to derive traveling wave solutions for the model. In another approach, [33] employed the projected differential transform method to obtain traveling wave patterns. Further, [34] concentrated on bifurcation and Painlevé singularity analysis within the Ivancevic option pricing framework. Other works, such as those in [35–37], focused exclusively on soliton solutions.

The concern with complexity involves the consideration of chaotic patterns, wave structures, Poincaré maps, bifurcation diagrams, multistability, Lyapunov exponents, and sensitivity within the Ivancevic option pricing model. Specifically, chaotic patterns are the basis for studying the stochastic nature of the system and its dependence on initial conditions [38]. From the analysis of the wave modes, we can learn how the phenomena propagate around the model; Poincaré maps and bifurcation diagrams are used as the primary instruments searching for the transitions between distinct states, for example, between periodic and chaotic ones. These diagrams not only define how the system’s parameters should be maintained for stability but also provide information on what bifurcations must occur to greatly change the system’s future performance.

Multistability and Lyapunov exponents offer additional quantitative characteristics of the stability of the system. Multistability provides ways to comprehend the existence of two or more stable states for the purpose of comprehending the impact of initial conditions and the potential outcomes. Lyapunov exponents, however, measure the rate of separation of the trajectories and hence provide a precise measure of chaos and stability in the system [39]. Sensitivity analysis builds upon this by showing how different parameter changes influence the dynamics of the system, allowing for models to be fine-tuned for better predictions as well as revealing outliers where slight differences in input can result in large alterations in behavior. Altogether these tools represent a rather comprehensive set of instruments to study the stability, chaos, and sensitivity issues in nonlinear models, which exist in finance, physics, and other sciences.

In this study, we looked at the IOPM from three different angles: Initially, a partial differential equation was transformed into an ordinary differential equation via a wave transformation. $\left(\frac{G^{\prime }}{G}\right)$ technique yielded the soliton solutions. In addition, suitable constant parameters are assigned to the calculated solutions, generating 2D and 3D graphs that demonstrate the corresponding physical events. After constructing a system of ordinary differential equations, we subsequently create an unperturbed dynamic system. We carry out an extensive examination of the qualitative features of the model through this dynamic system. Our research contains a thorough analysis that incorporates Lyapunov exponents, 2D phase portraits, time series graphs, Poincaré maps, multistability, wave structures, and chaos theory concepts. Finally, we evaluated at the suggested model’s sensitivity to different points of departure. Minor modifications to the starting points can result in a minor change in the system’s outcome. As a result, our observation implies that the recommended system shows some sensitivity, albeit not a great deal.

The paper is structured as follows: First, we derive the solitary wave solutions of the IOPM using the $\left(\frac{G}{G^{\prime }}\right)$ method and provide a graphical analysis. Additionally, 3D and 2D graphs are produced to illustrate the physical behavior of the obtained solutions by assigning suitable constant parameters. Next, we conduct a comprehensive sensitivity analysis of the initial conditions, followed by an exploration of quasi-periodic and chaotic behaviors in response to external perturbations. A multistability analysis is then performed concerning initial conditions. To further investigate chaotic motions within the model, we present a bifurcation diagram. This is complemented by a discussion of wave solutions and the use of the Poincaré section to detect chaotic behavior. Additionally, we analyze the Lyapunov characteristic exponent to confirm the presence of chaos. Finally, we summarize the key findings and discuss potential future research directions.

## Option-pricing wave function

In this part, we explore the option-pricing wave function for Eq (3). We define a transformation of the form:

$$\begin{array}{c} \Theta \left(S,\tau \right)=Y\left(\xi \right)e^{\iota \phi }, \end{array}$$ (4)

here, *Y*(*ξ*) represents the configuration of the wave pattern:

$$\begin{array}{c} \xi \left(S,\tau \right)=z_{0}\tau +z_{1}S. \end{array}$$ (5)

In this respect *z*_0_ plays the role of a coefficient that modulates the time variable and *z*_1_ is the rate of the soliton pulse. The phase component has been expressed as:

$$\begin{array}{c} \phi \left(S,\tau \right)=z_{3}\tau +z_{4}S. \end{array}$$ (6)

Here, *z*_3_ is the frequency range, and *z*_4_ is the wavelength number. By connecting Eqs (5) and (6) into Eq (4), we get the following mathematical structure:

$$\begin{array}{c} z_{2}^{2}Y^{\prime \prime }-\left(z_{4}^{2}+2z_{3}\right)Y+2\beta_{0}Y^{3}+i\left(z_{0}+z_{4}z_{2}\right)=0. \end{array}$$ (7)

By setting the imaginary part of Eq (7) equal to zero, we obtain:

$$\begin{array}{c} z_{0}=-z_{4}z_{2}. \end{array}$$ (8)

By setting the Real part of Eq (7) equal to zero, we obtain:

$$\begin{array}{c} z_{2}^{2}Y^{\prime \prime }-\left(z_{4}^{2}+2z_{3}\right)Y+2\beta_{0}Y^{3}=0 \end{array}$$ (9)

Substituting the value of *z*_4_ from Eqs (8) into (9), we have:

$$\begin{array}{c} z_{2}^{4}Y^{\prime \prime }-\left(z_{0}^{2}+2z_{3}z_{2}^{2}\right)Y+2z_{2}^{2}\beta_{0}Y^{3}=0. \end{array}$$ (10)

We apply the $\left(\frac{G^{\prime }}{G}\right)$ method on Eq (10) to derive the solitary option-pricing wave solution of Eq (3), with detailed procedures provided in [40]. By balancing *Y*^3^ and *Y*″ in Eq (10), we get *U*_0_ = 1. The solution for Eq (10) is as outlined below:

$$\begin{array}{c} Y\left(\xi \right)=\sum_{q_{0}=0}^{U_{0}}v_{q_{0}}(\frac{G^{\prime }}{G}{)}^{q_{1}}+\sum_{q_{1}=1}^{U_{0}}v_{q_{1}}^{⋆}(\frac{G^{\prime }}{G}{)}^{-q_{1}}, \end{array}$$ (11)

where *G* = *G*(*ξ*) fulfils:

$$\begin{array}{c} G^{\prime \prime }=\theta_{1}G^{\prime }+\theta_{2}\frac{{\left(G^{\prime }\right)}^{2}}{G}+\theta_{3}G. \end{array}$$ (12)

By inserting Eqs (11) into (10), we get a system of algebraic equations. Utilizing Maple, we can address algebraic equations to gain:

$$\begin{array}{c} \left\{ \begin{array}{c} \begin{array}{cc} & v_{1}=\frac{2v_{0}\left(\theta_{1}-1\right)}{\theta_{2}},\;\;\;v_{0}=v_{0},\;\;v_{1}^{⋆}=0,\;\;\beta_{0}=\frac{-\theta_{2}^{2}z_{2}^{2}}{4w_{0}^{2}},\\ & z_{3}=\frac{4\theta_{1}\theta_{3}z_{2}^{4}-\theta_{2}^{2}z_{2}^{4}-4\theta_{3}z_{2}^{4}-2z_{1}^{2}}{z_{2}^{2}},\;\;z_{4}=z_{4}. \end{array} \end{array}\right. \end{array}$$ (13)

Substituting the values into Eq (11) and applying Eq (4), the solutions to Eq (10) and Eq (3) are obtained as follows.

• The solution in terms of hyperbolic functions is valid when Γ_1_ = *θ*_2_ − 4*θ*_3_(*θ*_1_ − 1) is greater than zero and *θ*_1_ ≠ 0:

$$\begin{array}{c} Y_{1}\left(\xi \right)=v_{0}-\frac{v_{0}\sqrt{\Gamma_{1}}}{\theta_{2}}(\frac{H_{1}^{⋆}\;\text{sinh}(\frac{\sqrt{\Gamma_{1}}\xi }{2})+H_{2}^{⋆}\;\text{cosh}(\frac{\sqrt{\Gamma_{1}}\xi }{2})}{H_{1}^{⋆}\;\text{cosh}(\frac{\sqrt{\Gamma_{1}}\xi }{2})+H_{2}^{⋆}\;\text{sinh}(\frac{\sqrt{\Gamma_{1}}\xi }{2})}-\frac{\theta_{2}}{2}), \end{array}$$ (14)

$$\begin{array}{c} \Theta_{1}\left(S,\tau \right)=e^{\iota (z_{0}\tau +z_{1}S)}(v_{0}-\frac{v_{0}\sqrt{\Gamma_{1}}}{\theta_{2}}(\frac{H_{1}^{⋆}\;\text{sinh}(\frac{\sqrt{\Gamma_{1}}}{2}\left(z_{0}\tau +z_{1}S\right))+H_{2}^{⋆}\;\text{cosh}(\frac{\sqrt{\Gamma_{1}}\left(z_{0}\tau +z_{1}S\right)}{2})}{H_{1}^{⋆}\;\text{cosh}(\frac{\sqrt{\Gamma_{1}}}{2}\left(z_{0}\tau +z_{1}S\right))+H_{2}^{⋆}\;\text{sinh}(\frac{\sqrt{\Gamma_{1}}\left(z_{0}\tau +z_{1}S\right)}{2})}-\frac{\theta_{2}}{2})). \end{array}$$ (15)

• The solution in terms of trigonometry functions is valid when Γ_1_ = *θ*_2_ − 4*θ*_3_(*θ*_1_ − 1) is less than zero and *θ*_1_ ≠ 0:

$$\begin{array}{c} Y_{2}\left(\xi \right)=v_{0}-\frac{v_{0}\sqrt{\Gamma_{1}}}{\theta_{2}}(\frac{-H_{1}^{⋆}\;\text{sin}(\frac{\sqrt{\Gamma_{1}}\xi }{2})+H_{2}^{⋆}\;\text{cos}(\frac{\sqrt{\Gamma_{1}}\xi }{2})}{H_{1}^{⋆}\;\text{cos}(\frac{\sqrt{\Gamma_{1}}\xi }{2})+H_{2}^{⋆}\;\text{sin}(\frac{\sqrt{\Gamma_{1}}\xi }{2})}-\frac{\theta_{2}}{2}), \end{array}$$ (16)

$$\begin{array}{c} \Theta_{2}\left(S,\tau \right)=e^{\iota (z_{0}\tau +z_{1}S)}(v_{0}-\frac{v_{0}\sqrt{\Gamma_{2}}}{\theta_{2}}(\frac{-H_{1}^{⋆}\;\text{sin}(\frac{\sqrt{\Gamma_{2}}}{2}\left(z_{0}\tau +z_{1}S\right))+H_{2}^{⋆}\;\text{cos}(\frac{\sqrt{\Gamma_{2}}\left(z_{0}\tau +z_{1}S\right)}{2})}{H_{1}^{⋆}\;\text{cos}(\frac{\sqrt{\Gamma_{2}}}{2}\left(z_{0}\tau +z_{1}S\right))+H_{2}^{⋆}\;\text{sin}(\frac{\sqrt{\Gamma_{2}}\left(z_{0}\tau +z_{1}S\right)}{2})}-\frac{\theta_{2}}{2}))\;\cdot \end{array}$$ (17)

• The solution in terms of rational functions is valid when *θ*_2_ − 4*θ*_3_(*θ*_1_ − 1) is equal to zero and *θ*_1_ ≠ 0:

$$\begin{array}{c} Y_{3}\left(S,\tau \right)=(\frac{-2v_{0}}{\theta_{2}}(\frac{H_{1}^{⋆}}{H_{1}^{⋆}\xi +H_{2}^{⋆}}+\frac{\theta_{2}}{2})), \end{array}$$ (18)

$$\begin{array}{c} \Theta_{3}\left(S,\tau \right)=e^{\iota (z_{0}\tau +z_{1}S)}(\frac{-2v_{0}}{\theta_{2}}(\frac{H_{1}^{⋆}}{H_{1}^{⋆}\left(z_{0}\tau +z_{1}S\right)+H_{2}^{⋆}}+\frac{\theta_{2}}{2}))\;. \end{array}$$ (19)

### Graphical representation of results

**Fig 1:**
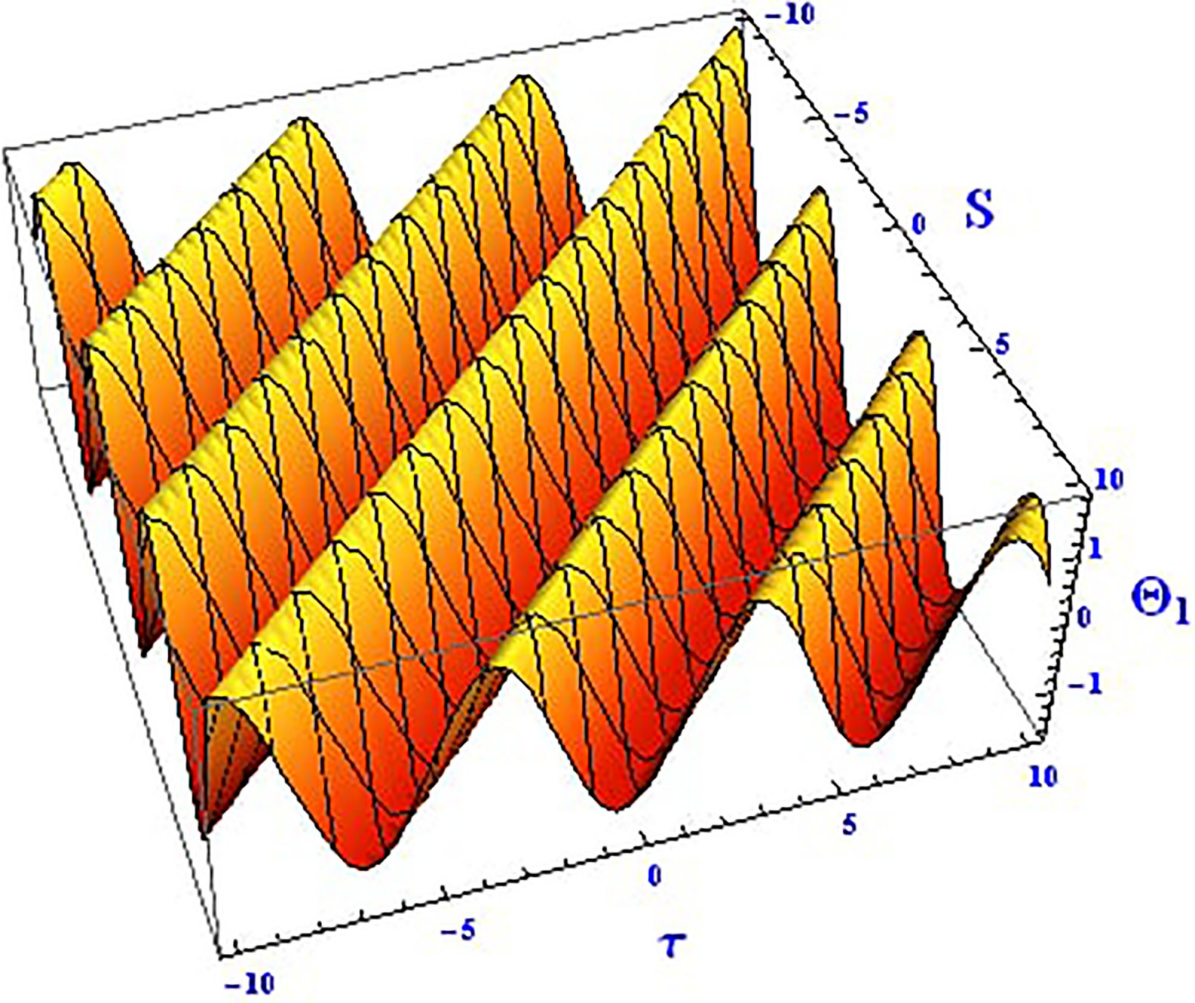
Graphical depiction of the 3D real solution of Θ_1_(*S*, *τ*) presented by Eq (3), showing a periodic pattern.

**Fig 2:**
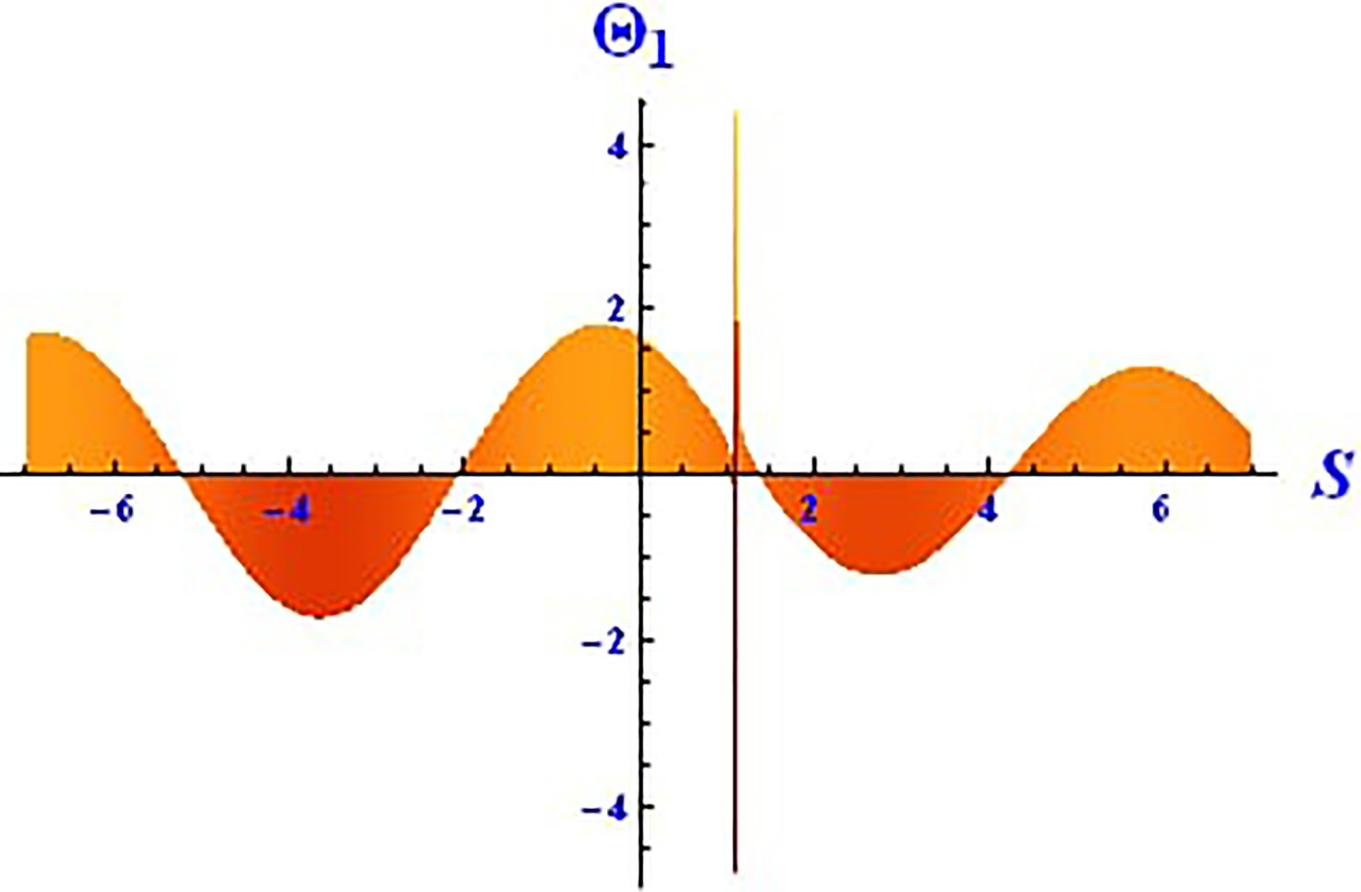
Graphical depiction of the 2D real solution of Θ_1_(*S*, *τ*) presented by Eq (3), showing a periodic pattern.

**Fig 3:**
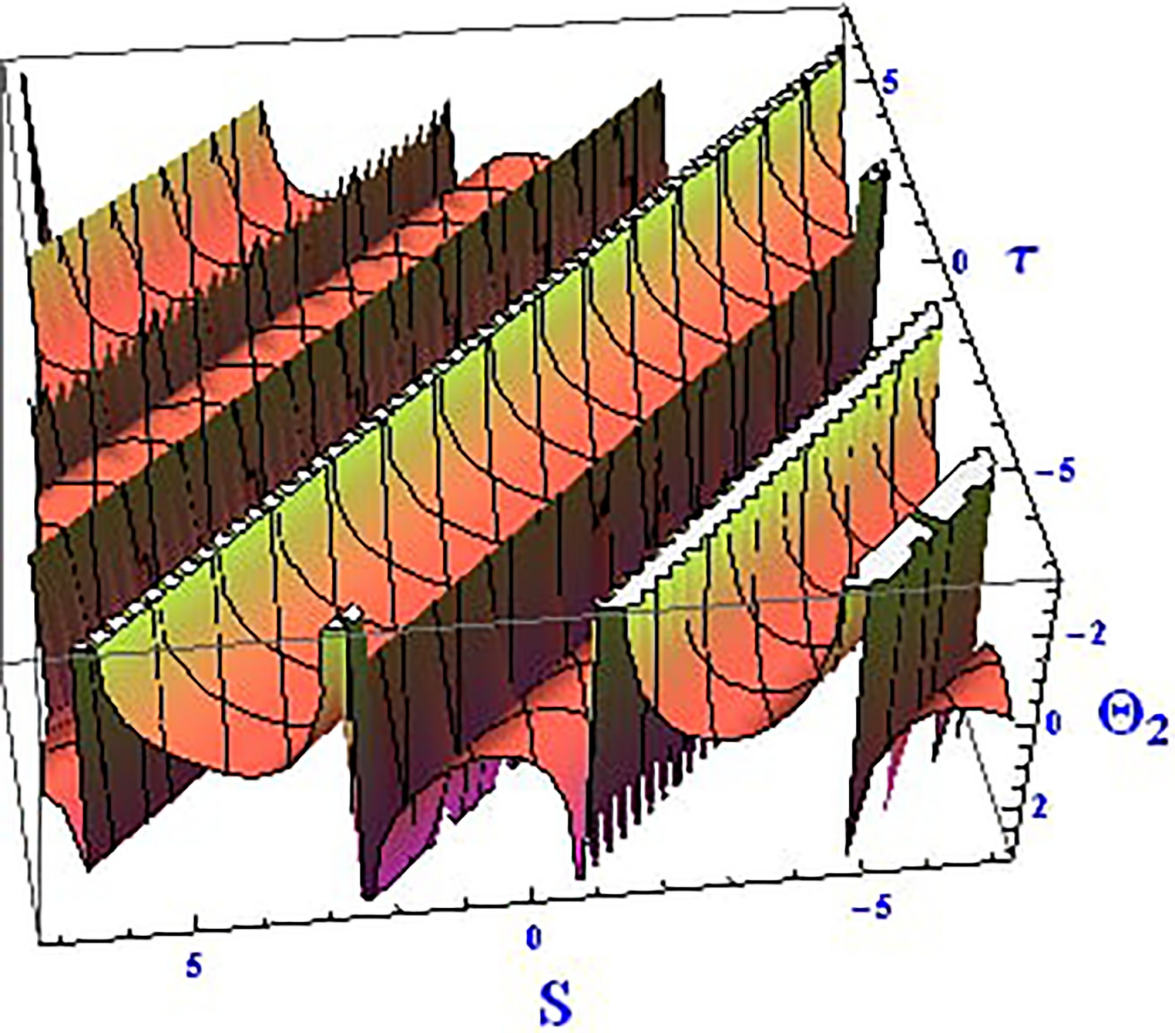
Graphical depiction of the 3D imaginary solution of Θ_1_(*S*, *τ*) presented by Eq (3), showing a singular periodic pattern.

**Fig 4:**
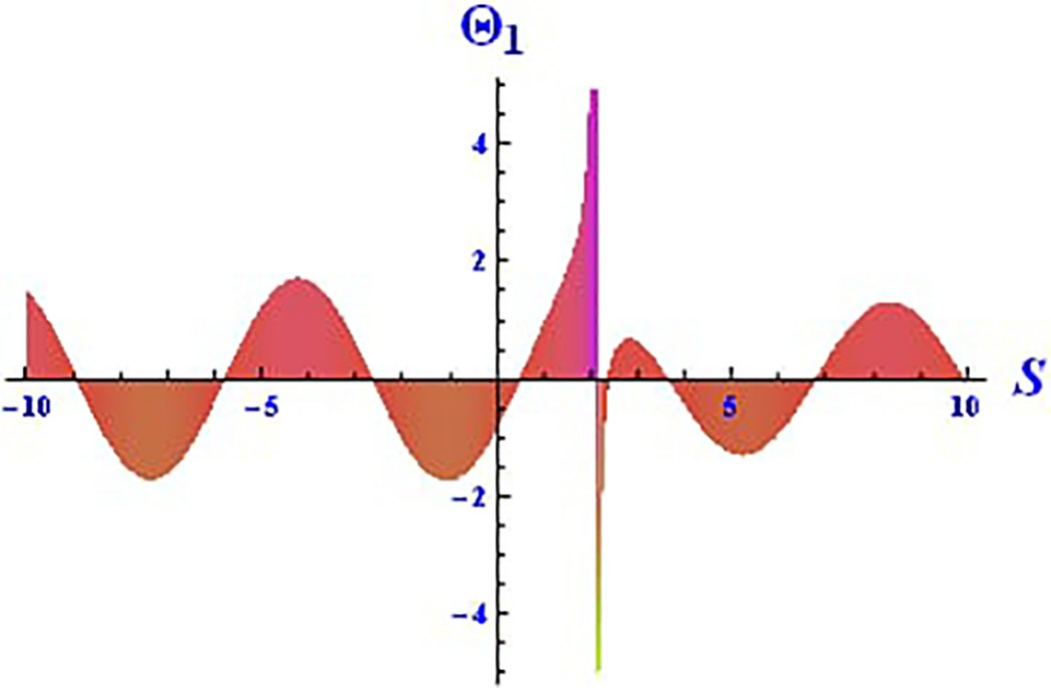
Graphical depiction of the 2D imaginary solution of Θ_1_(*S*, *τ*) presented by Eq (3), showing a singular periodic pattern.

**Fig 5:**
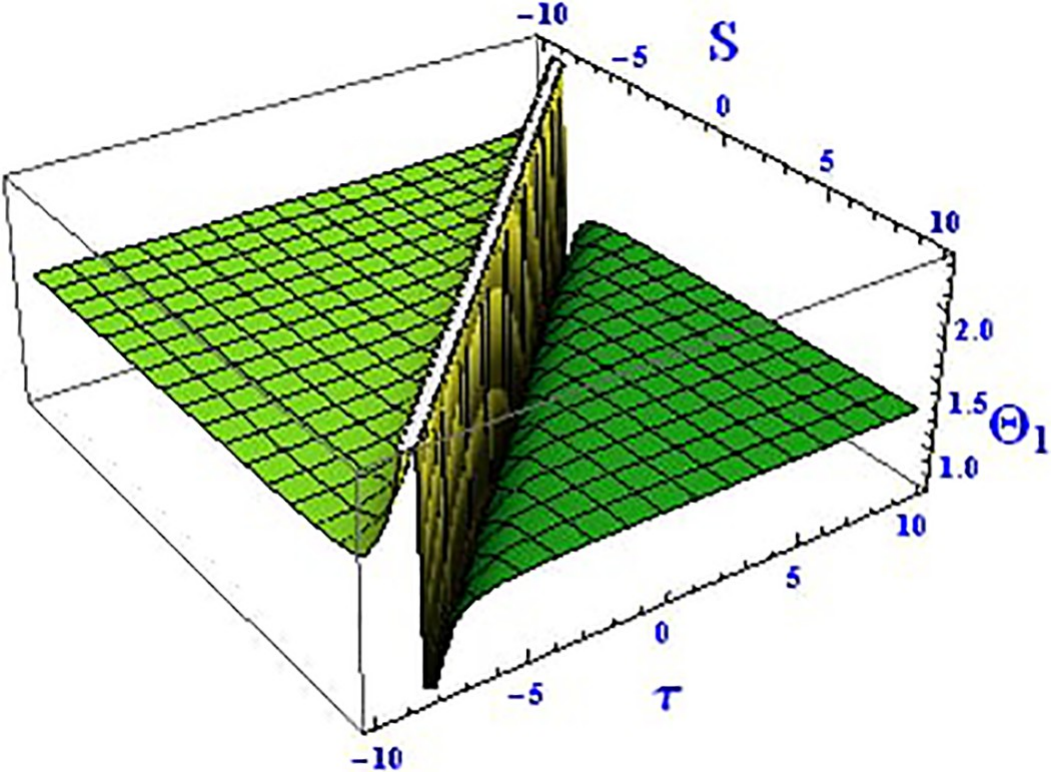
Graphical depiction of the 3D modulus solution of Θ_1_(*S*, *τ*) presented by Eq (3), showing a singular kink.

**Fig 6:**
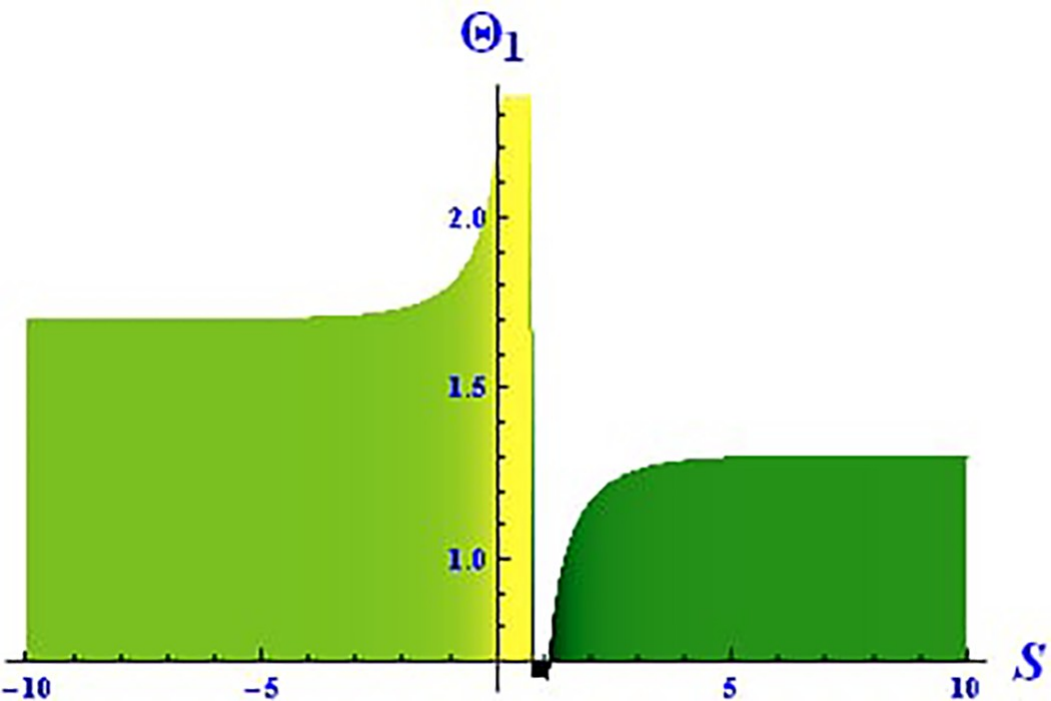
Graphical depiction of the 2D modulus solution of Θ_1_(*S*, *τ*) presented by Eq (3), showing a singular kink type.

Plotting wave solutions are useful for illustrating the internal structure of non-linear processes from the perspective of potential users. In this context, we present the obtained wave solutions in both 3D and 2D forms for the real part, imaginary part, and modulus profile with fixed parameters. The 2D and 3D plots of the real wave solution for Eq (15) with *θ*_2_ = 5, *θ*_1_ = 2, $H_{1}^{⋆}=2$, $H_{2}^{⋆}=-3$, and all other parameters set to one, within the domain −10 < *S*, *τ* < 10, are shown in Figs 1 and 2. The 2D and 3D wave solutions of the imaginary wave, corresponding to Eq (15) with *θ*_2_ = 5, *θ*_1_ = 2, $H_{1}^{⋆}=2$, $H_{2}^{⋆}=-3$, and other parameters set to one, within the domain −10 < *S*, *τ* < 10, are shown in Figs 3 and 4. The *S* = 1 profile is represented in the 2D plot, showcasing a singular periodic soliton pattern within −10 < *S*, *τ* < 10. The 2D and 3D modulus wave solutions are represented as singular kink-type solitons of the solution in Eq (15), as illustrated in Figs 5 and 6 with *θ*_1_ = 2, $H_{1}^{⋆}=2$, $H_{2}^{⋆}=-3$, and other parameters set to one, within −10 < *S*, *τ* < 10.

**Fig 7:**
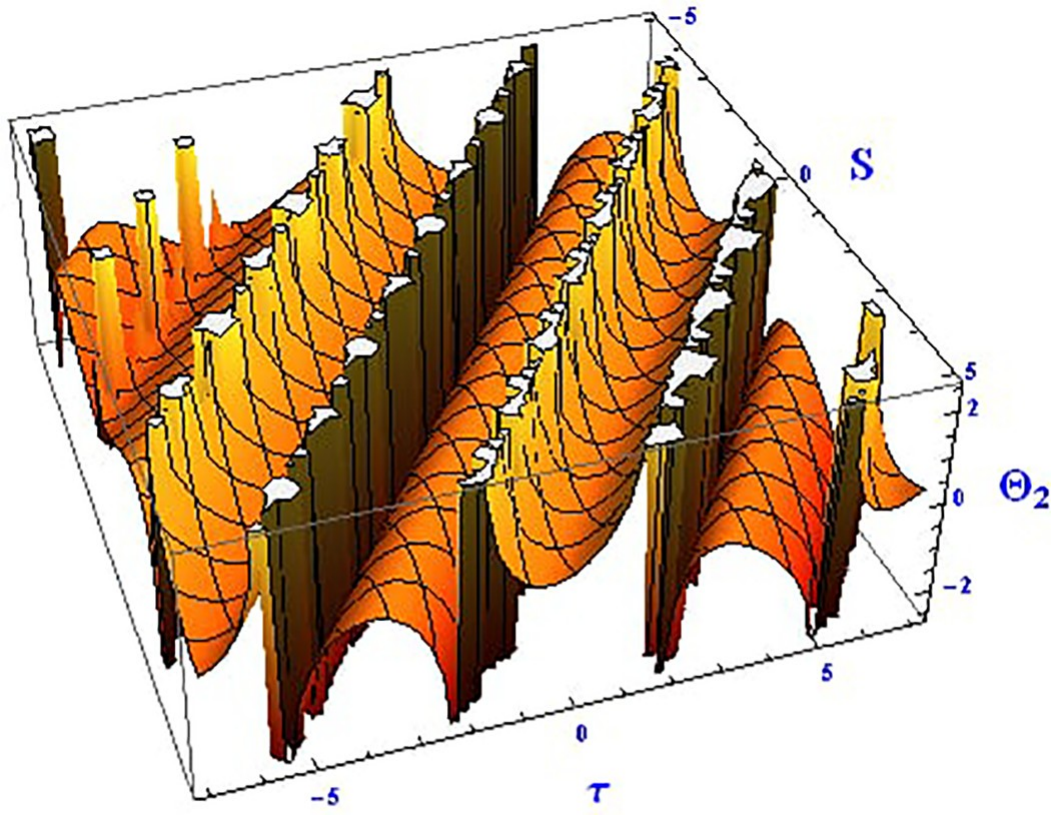
Graphical depiction of the 3D real solution of Θ_2_(*S*, *τ*) presented by Eq (3), showing a singular periodic pattern.

**Fig 8:**
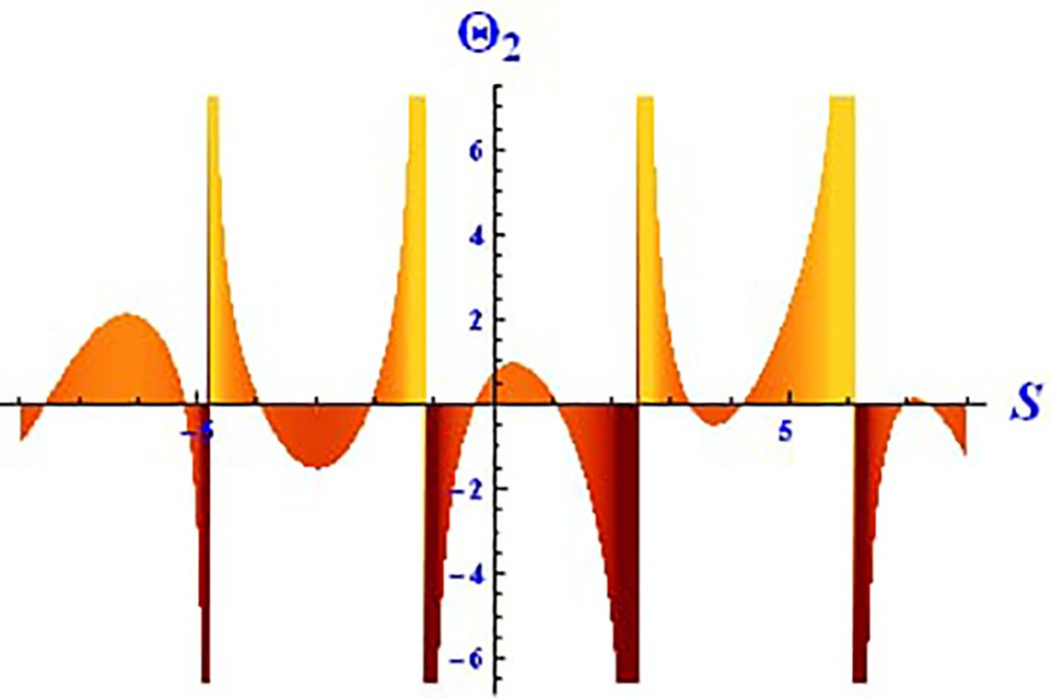
Graphical depiction of the 2D real solution of Θ_2_(*S*, *τ*) presented by Eq (3), showing a singular periodic pattern.

**Fig 9:**
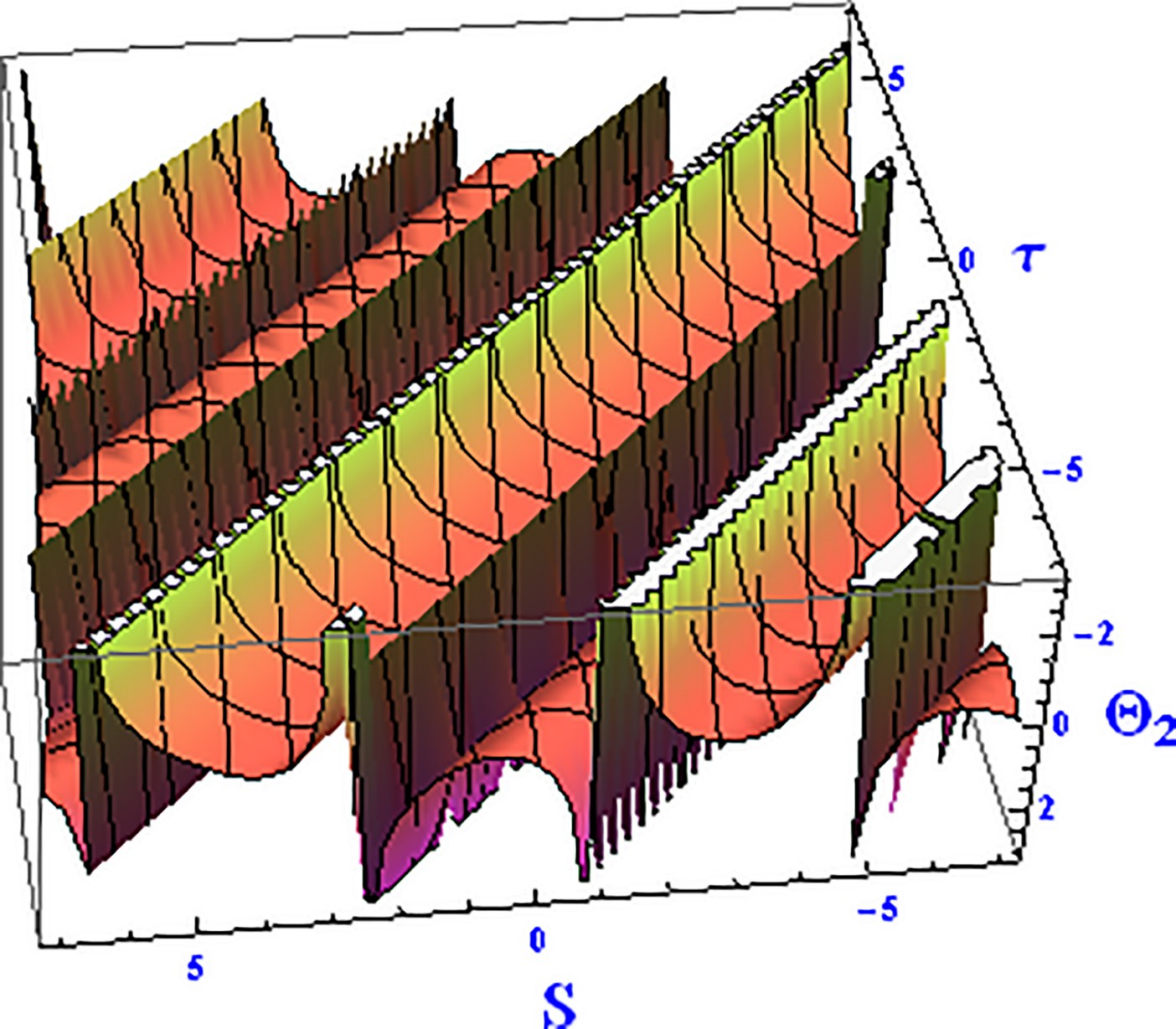
Graphical depiction of the 3D imaginary solution of Θ_2_(*S*, *τ*) presented by Eq (3), showing a singular periodic pattern.

**Fig 10:**
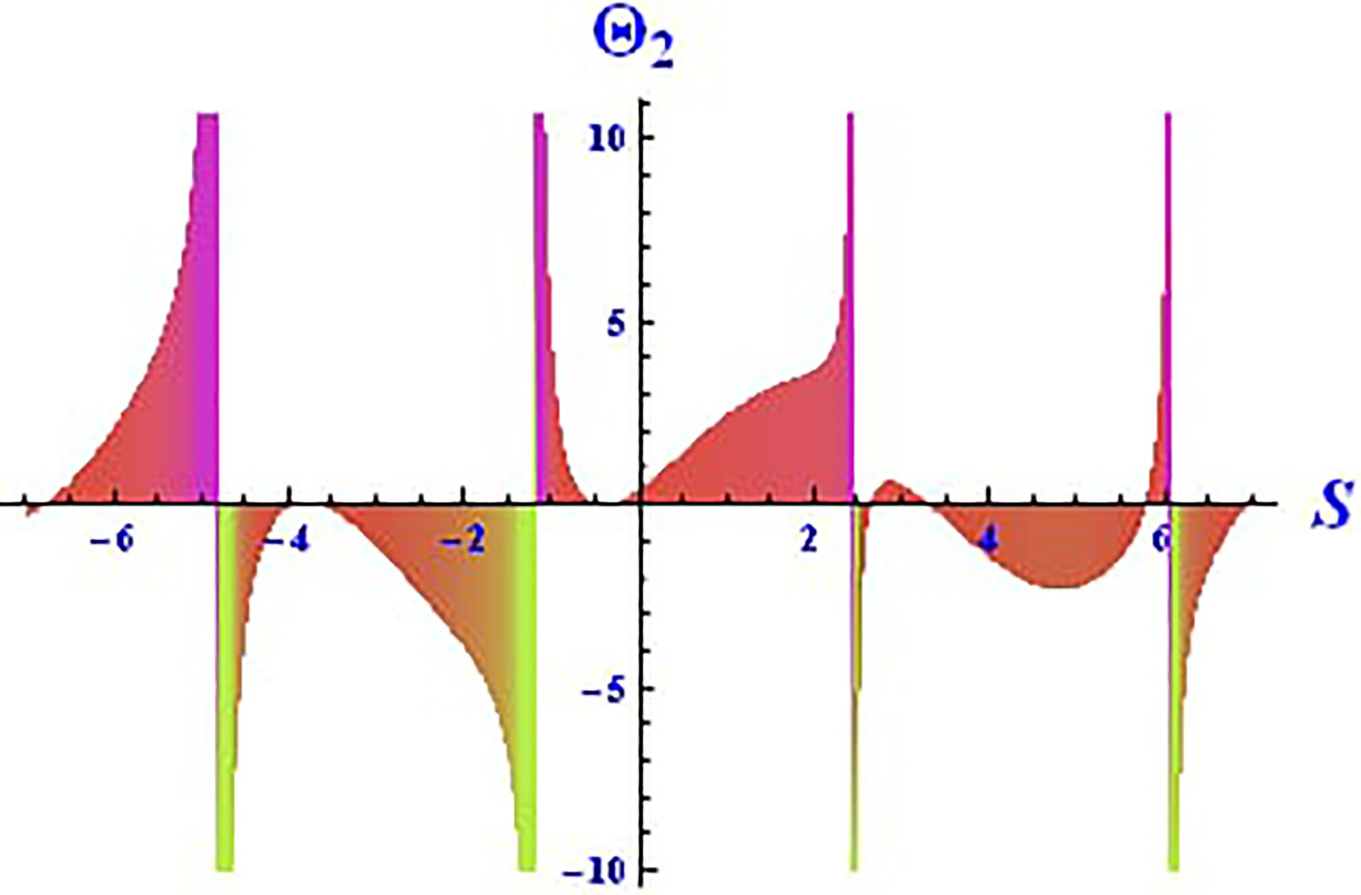
Graphical depiction of the 2D imaginary solution of Θ_2_(*S*, *τ*) presented by Eq (3), showing a singular periodic pattern.

**Fig 11:**
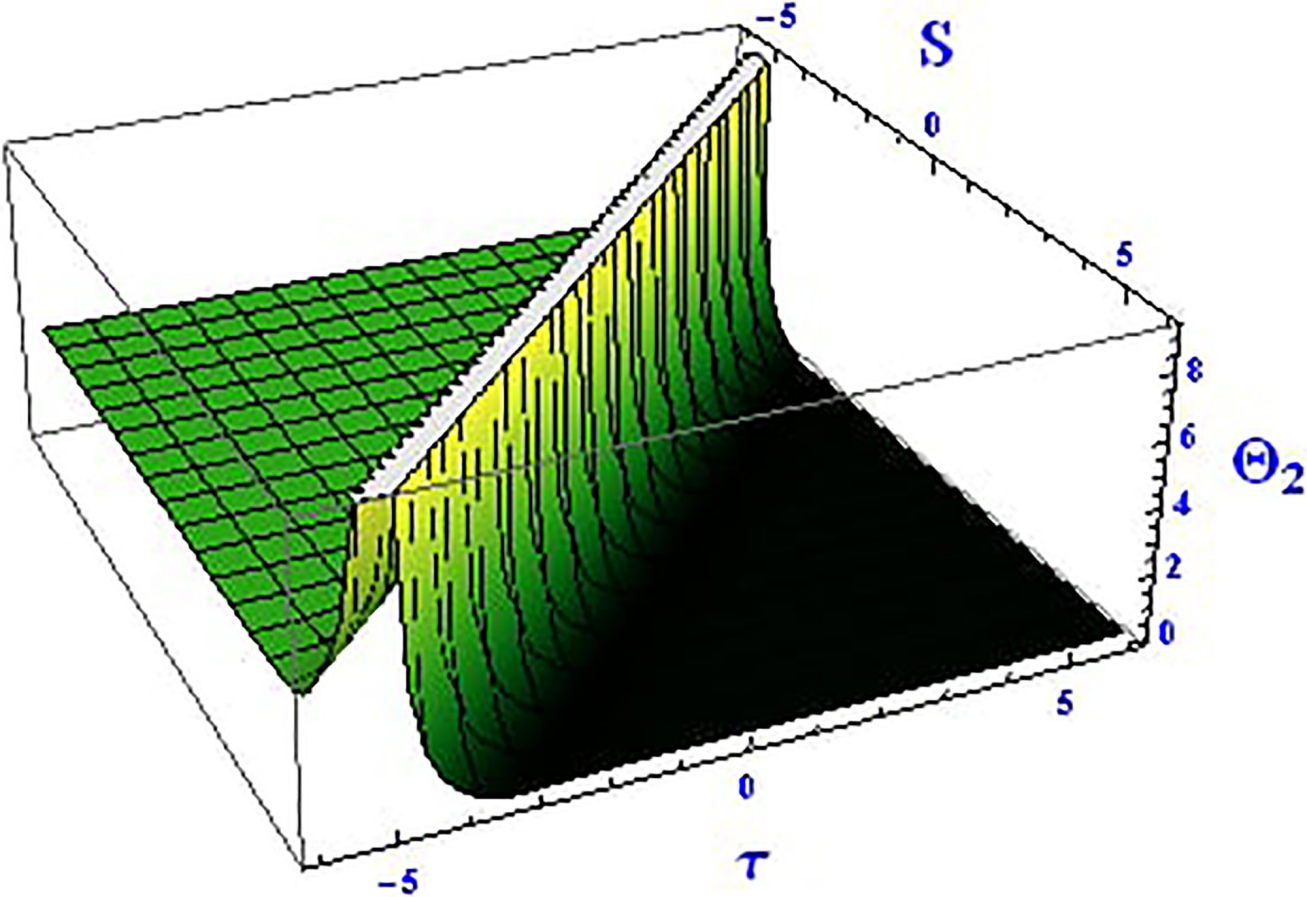
Graphical depiction of the 3D modulus solution of Θ_2_(*S*, *τ*) presented by Eq (3), showing a singular bell type soliton.

**Fig 12:**
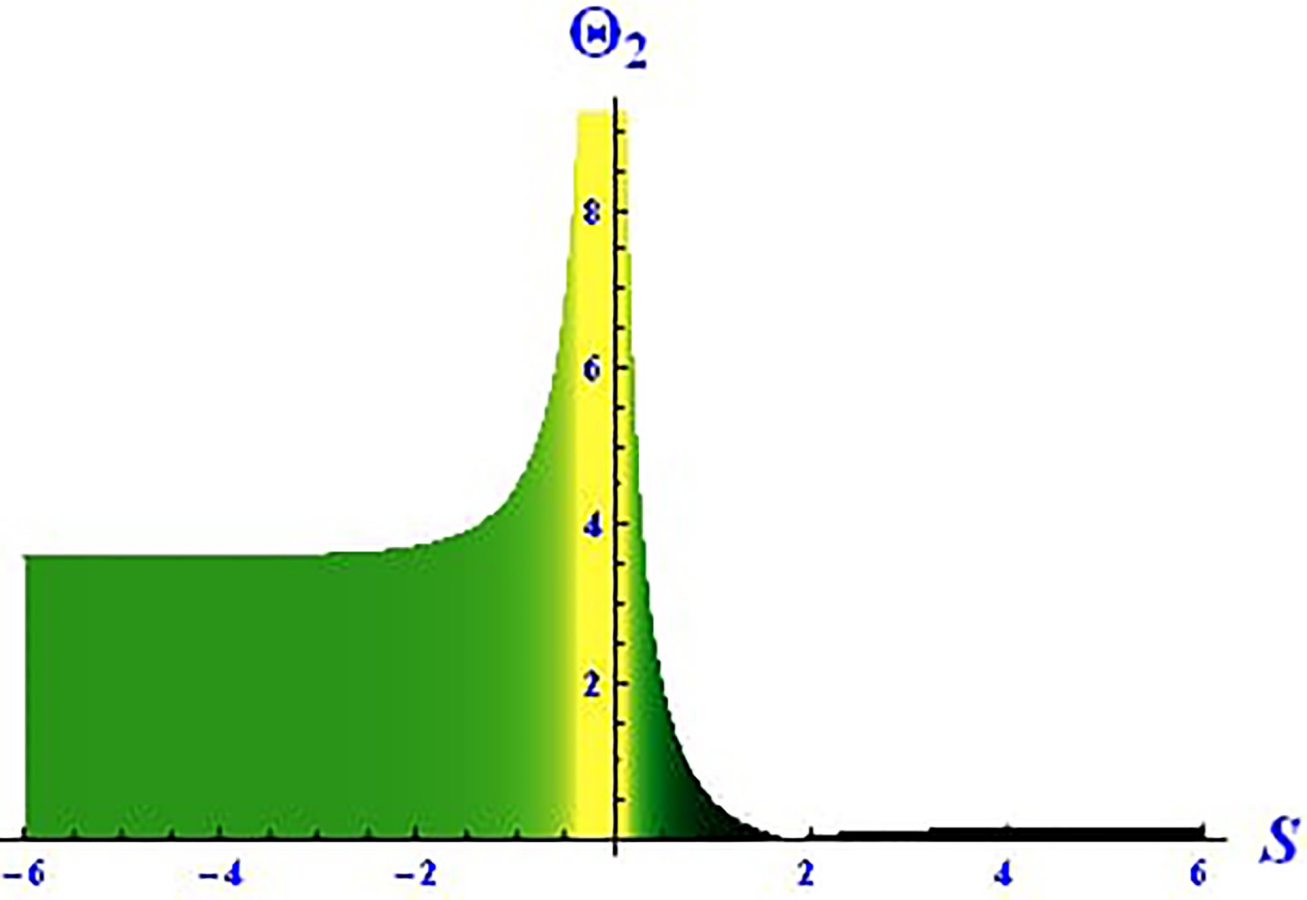
Graphical depiction of the 2D modulus solution of Θ_2_(*S*, *τ*) presented by Eq (3), showing a singular bell type soliton.

For the solution (17), the real wave is investigated under three cases: *θ*_2_ = 4, *θ*_1_ = 2, $H_{1}^{⋆}=2$, $H_{2}^{⋆}=-3$, and all other parameters set to one, as shown in Figs 7 and 8. These figures depict the 2D and 3D soliton profiles of the real wave, and the 2D plot illustrates the profile at *S* = 1 within the domain −10 < *S*, *τ* < 10. In Figs 9 and 10, the separated 2D and 3D wave solutions of the imaginary wave are shown for the solution in Eq (17) with *θ*_2_ = 4, *θ*_1_ = 2, $H_{1}^{⋆}=2$, $H_{2}^{⋆}=-3$, and all other parameters set to one. The figures display singular periodic soliton patterns within −10 < *S*, *τ* < 10. In Figs 11 and 12 presents the 2D and 3D modulus wave solutions as singular bell-type solitons of the solution in Eq (17) within the range −10 < *S*, *τ* < 10.

**Fig 13:**
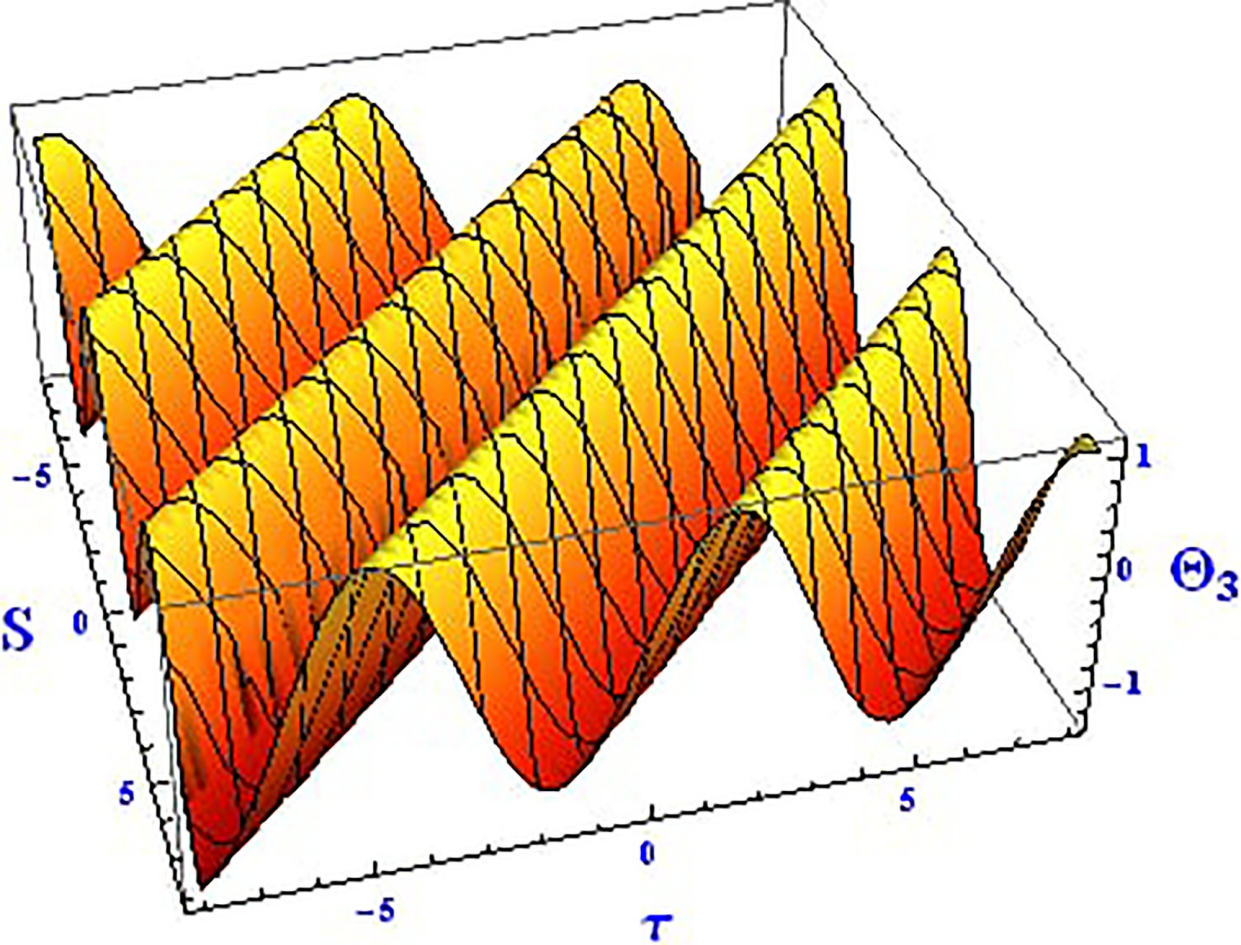
Graphical depiction of the 3D real solution of Θ_3_(*S*, *τ*) presented by Eq (3), showing a periodic pattern.

**Fig 14:**
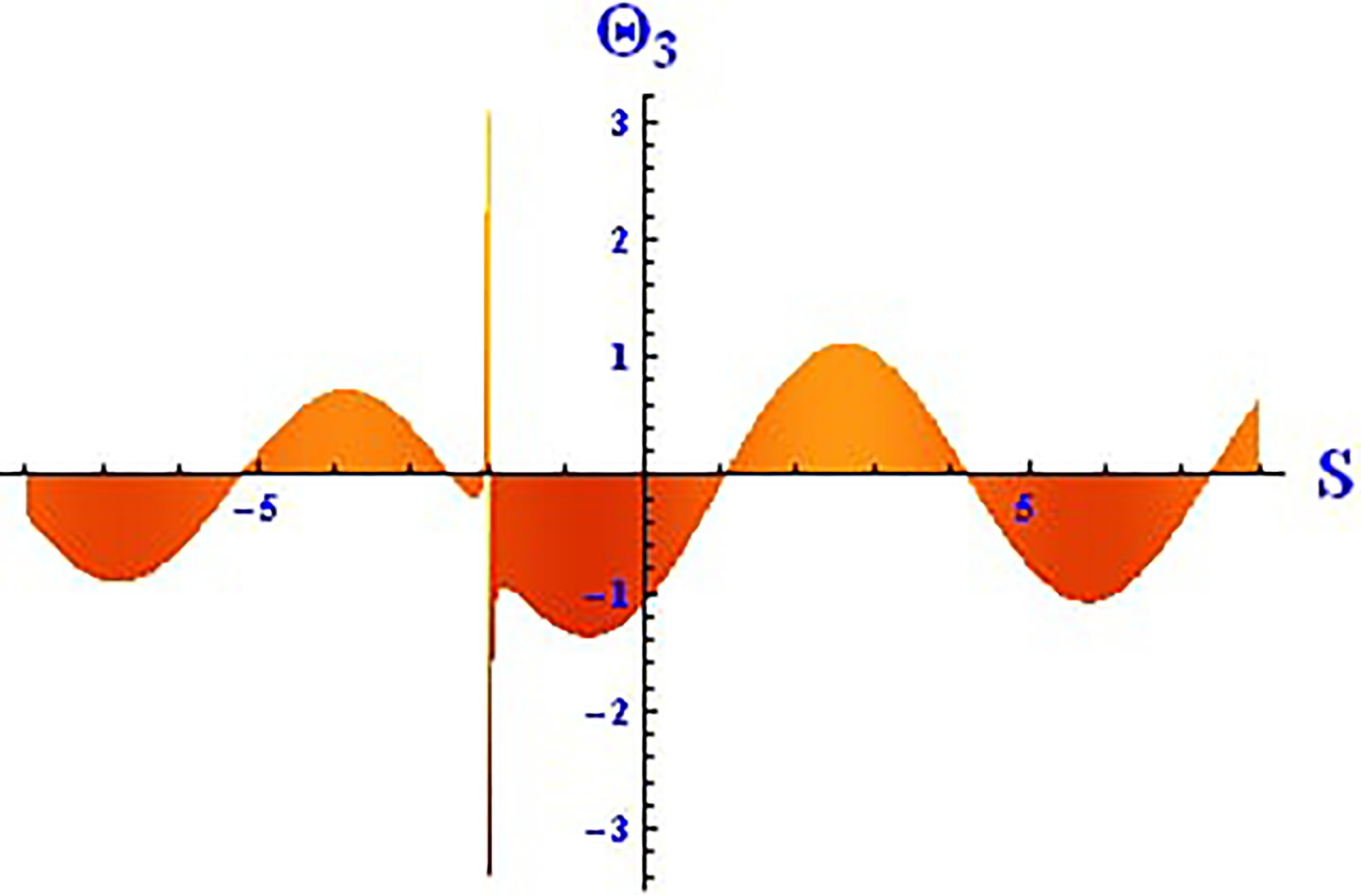
Graphical depiction of the 2D real solution of Θ_3_(*S*, *τ*) presented by Eq (3), showing a periodic pattern.

**Fig 15:**
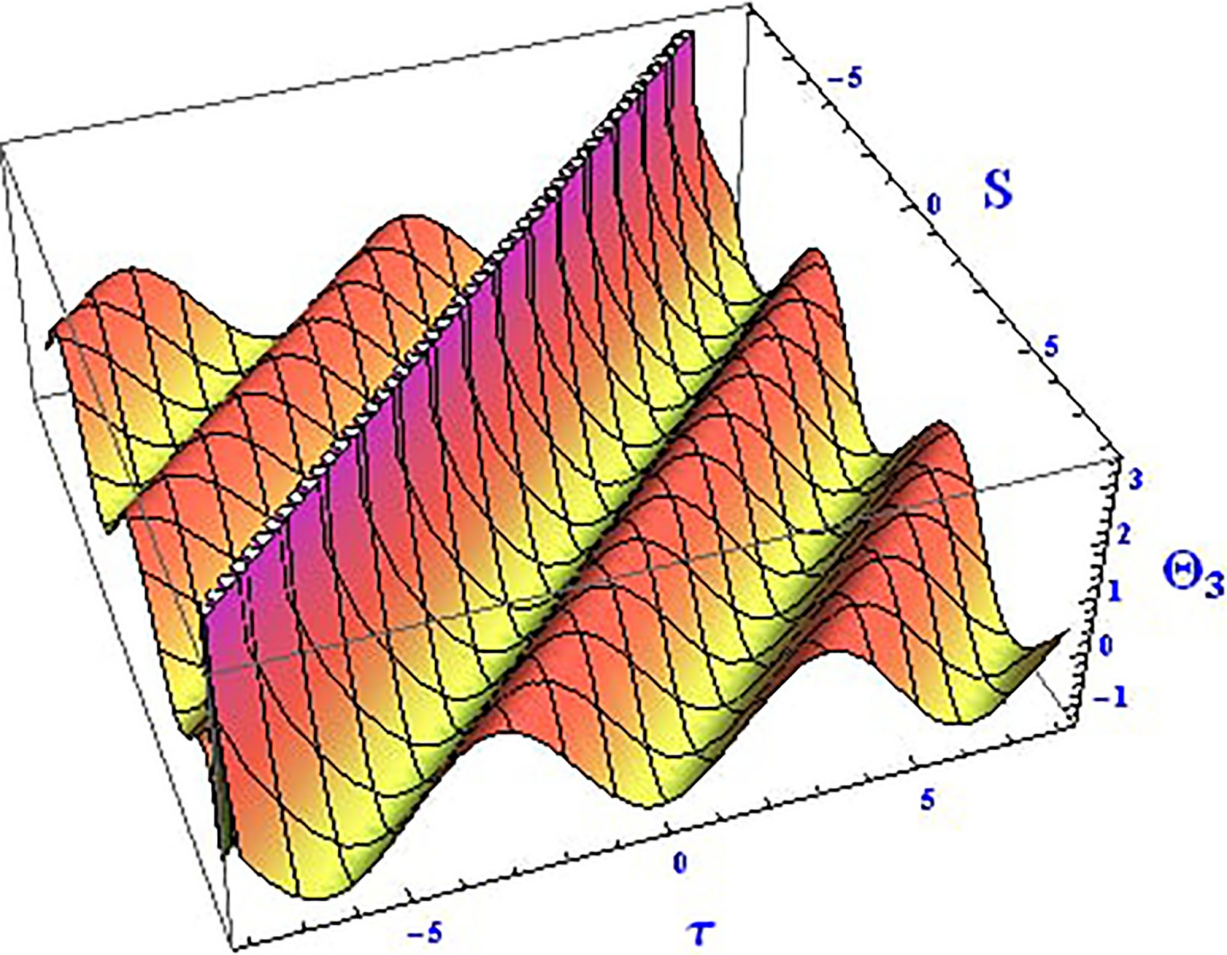
Graphical depiction of the 3D imaginary solution of Θ_3_(*S*, *τ*) presented by Eq (3), showing a periodic pattern.

**Fig 16:**
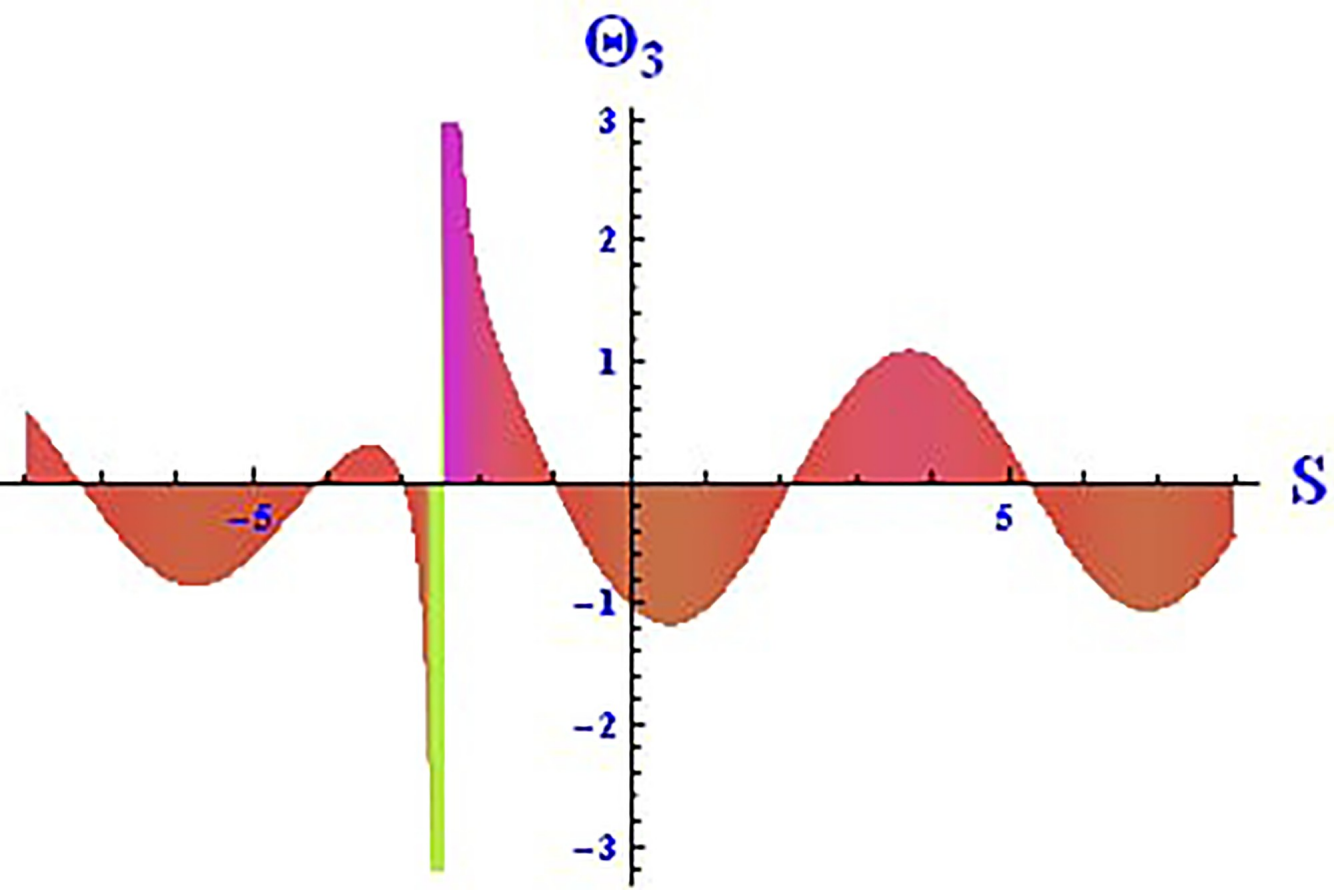
Graphical depiction of the 2D imaginary solution of Θ_3_(*S*, *τ*) presented by Eq (3), showing a periodic pattern.

The 2D and 3D solitons of the real wave solution for Eq (19) with *θ*_2_ = 4, *θ*_1_ = 2, $H_{1}^{⋆}=2$, $H_{2}^{⋆}=-3$, and all other parameters set to one, within the domain −10 < *S*, *τ* < 10, are depicted in Figs 13 and 14. The *S* = 1 profile is presented in the 2D plot. In Figs 15 and 16, the 2D and 3D wave solutions of the imaginary wave, corresponding to Eq (19) with *θ*_2_ = 4, *θ*_1_ = 2, $H_{1}^{⋆}=2$, $H_{2}^{⋆}=-3$, and all other parameters set to one, are displayed. Singular periodic soliton patterns within −10 < *S*, *τ* < 10 are also shown, with the *S* = 1 profile depicted in the 2D plot.

**Fig 17:**
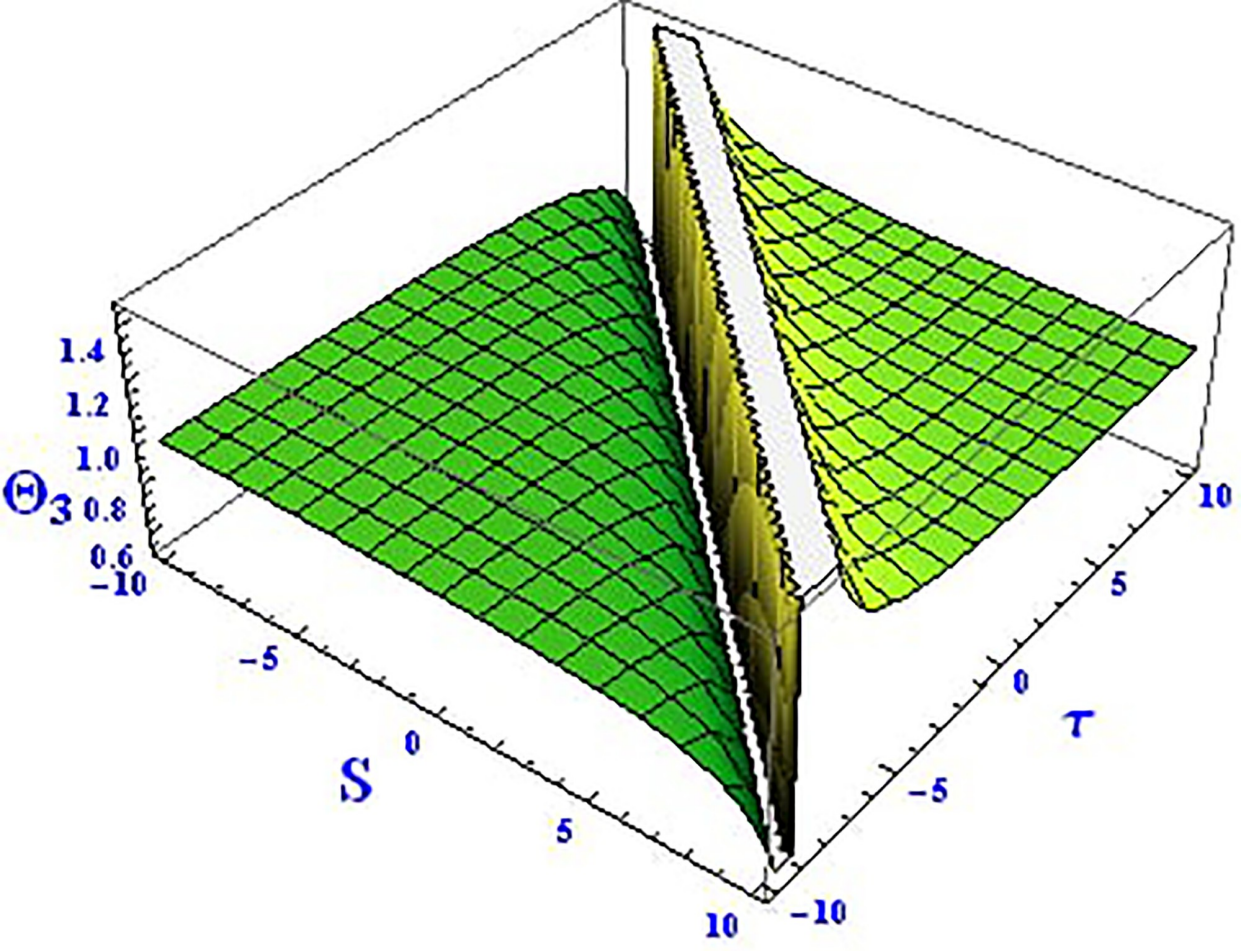
Graphical depiction of the 3D modulus solution of Θ_3_(*S*, *τ*) presented by Eq (3), showing a singular kink type solution.

**Fig 18:**
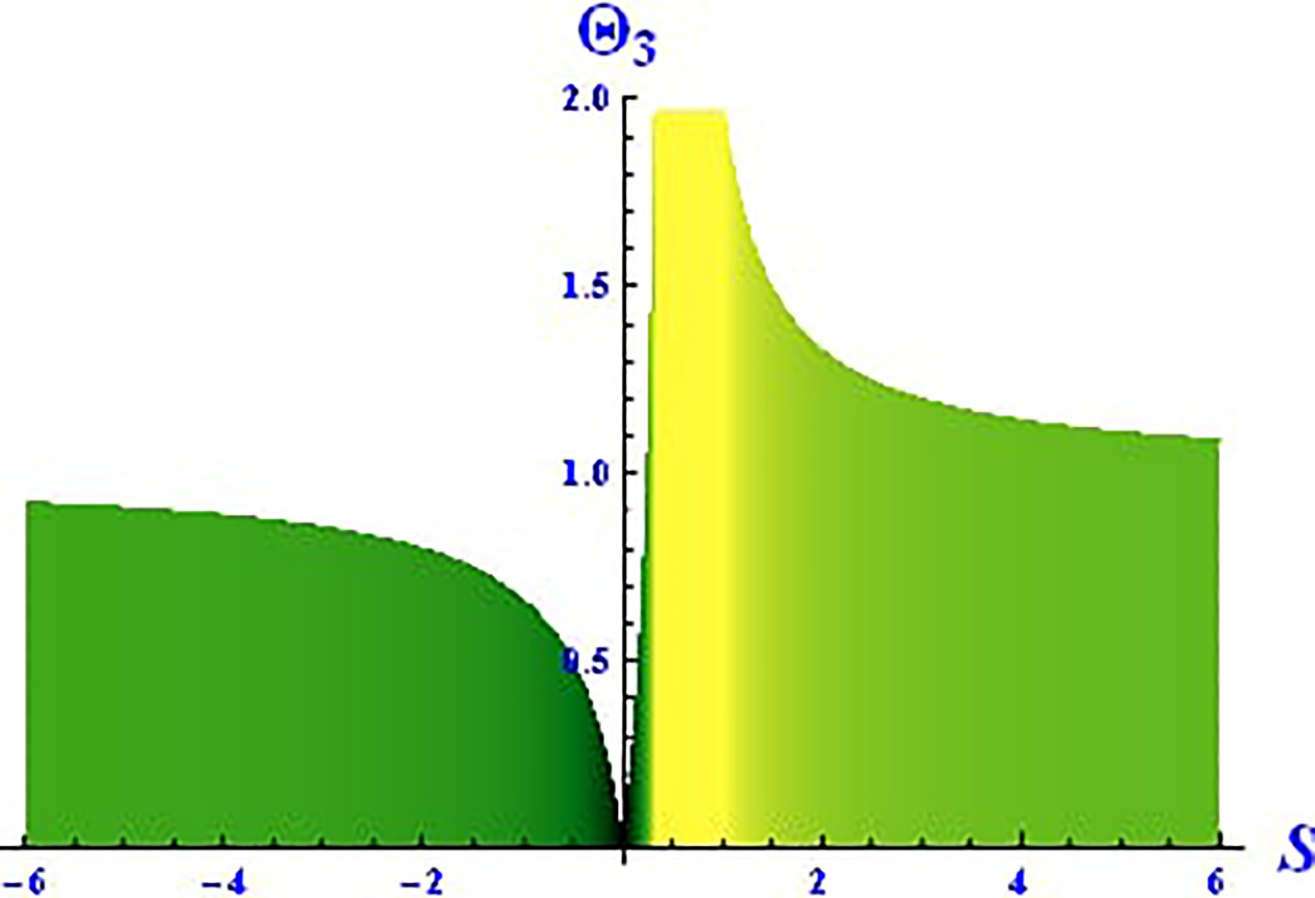
Graphical depiction of the 2D modulus solution of Θ_3_(*S*, *τ*) presented by Eq (3), showing a singular kink type solution.

Lastly, the 2D and 3D modulus wave solutions are represented as singular kink-type solitons for the solution in Eq (19), as depicted in Figs 17 and 18, with *θ*_2_ = 4, *θ*_1_ = 2, $H_{1}^{⋆}=2$, $H_{2}^{⋆}=-3$, and other parameters set to one, within −10 < *S*, *τ* < 10. The non-linear parameters, including the Landau coefficient, have a strong influence on the system.

## Sensitivity analysis

In this segment, we discuss the influence of the initial conditions on the governing equation of the problem. Two distinct initial conditions are taken into account to check the model sensitivity. Eq (10) can be converted into the following first-order differential equations using the Galilean transformation [41]:

$$\begin{array}{c} \left\{ \begin{array}{cc} & \frac{dY}{d\xi }=Z,\\ & \frac{dZ}{d\xi }=\alpha_{1}Y-\alpha_{2}Y^{3},\\ & \alpha_{1}=\frac{z_{0}^{2}+2z_{3}z_{2}^{2}}{z_{2}^{4}}\;,\;\;\alpha_{2}=\frac{2\beta_{0}}{z_{2}^{2}}. \end{array}\right. \end{array}$$ (20)

**Fig 19:**
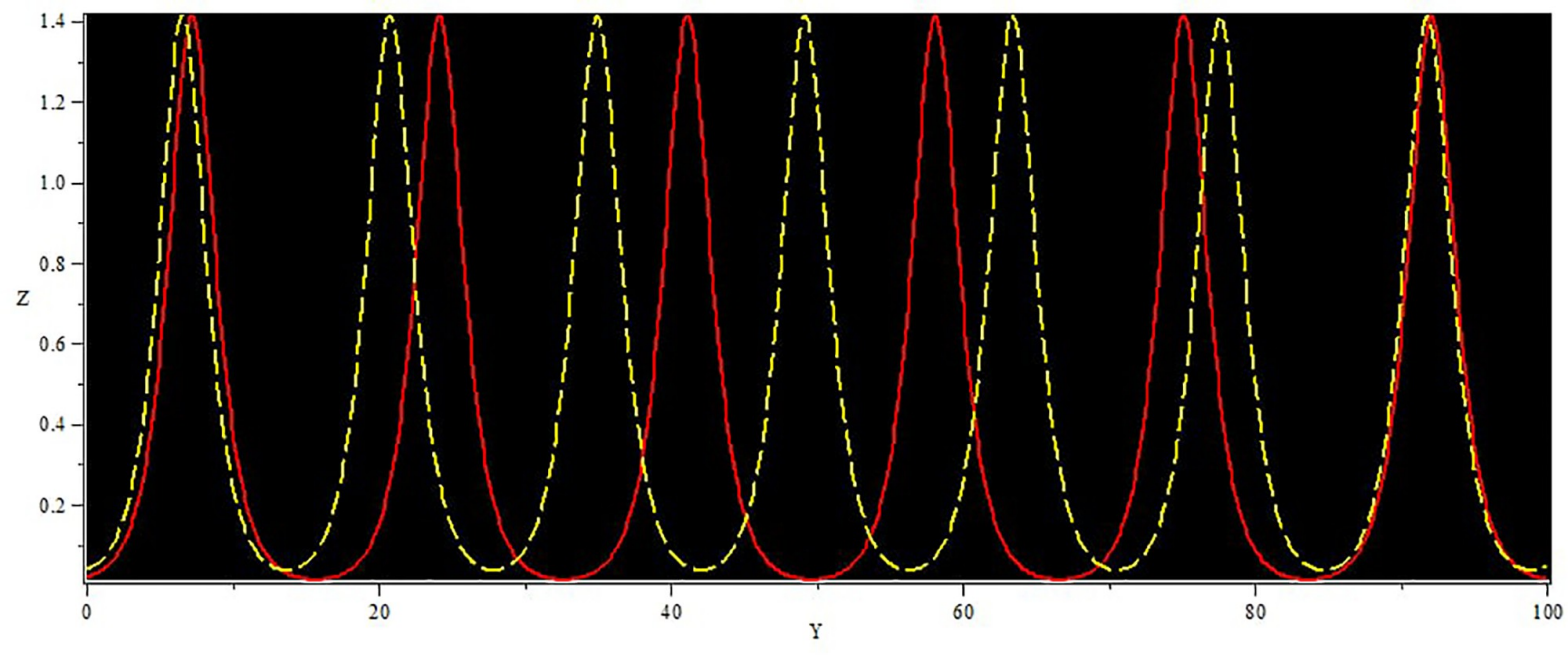
Sensitivity behaviour of the dynamical system (20) with Initial condition (0.02, 0.01) and (0.04, 0.01).

**Fig 20:**
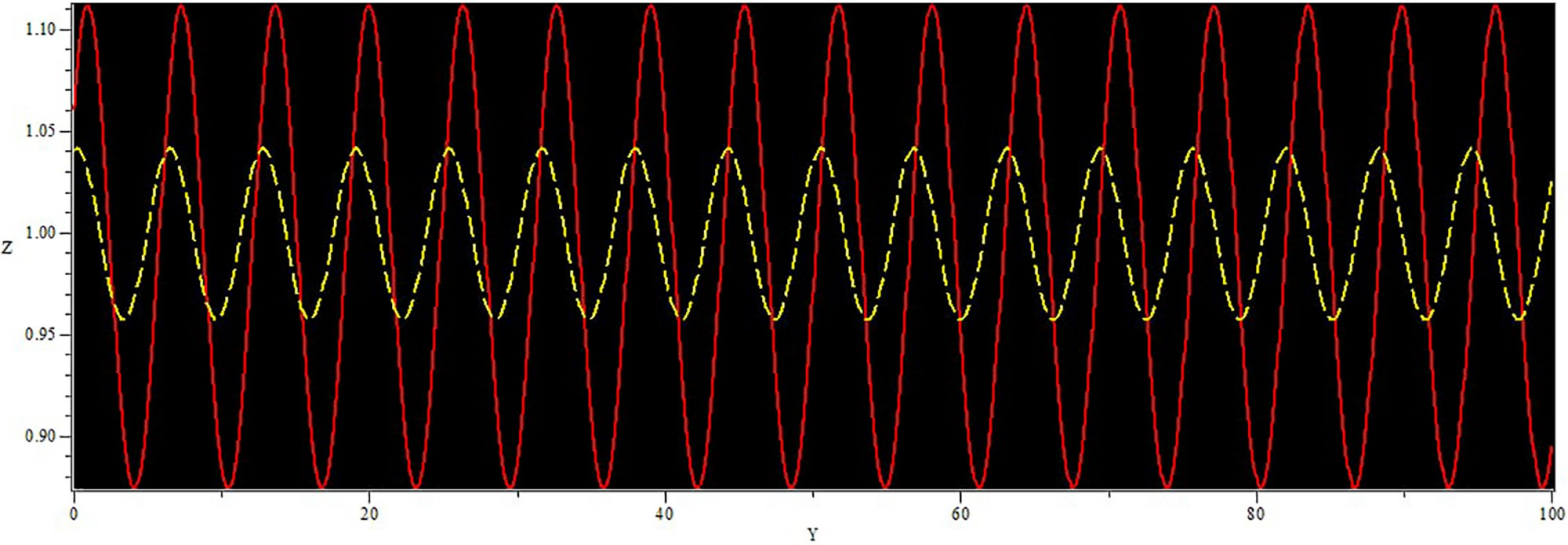
Sensitivity behavior of the dynamical system (20) with Initial condition (1.60, 0.01) and (1.50, 0.01).

**Fig 21:**
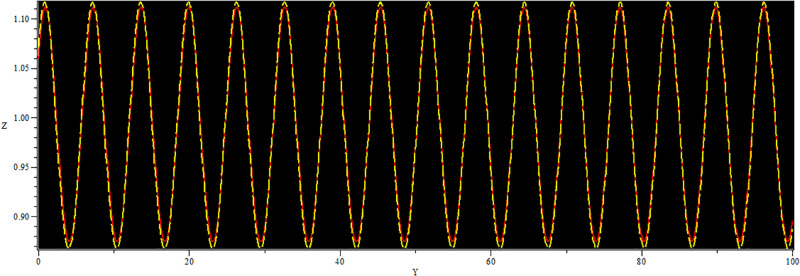
Sensitivity behaviour of the dynamical system (20) with Initial condition (1.60, 0.01) and (1.70, 0.01).

In Fig 19, two solutions are shown: The first one corresponds to the initial condition in the red curve is (Y, Z) = (0.02, 0.01), and the second set, in the yellow curve, is (Y, Z) = (0.04, 0.01). In Fig 20, we observe two solutions: first for the initial condition (*Y*, *Z*) = (1.50, 0.01) with a color red and second for (*Y*, *Z*) = (1.60, 0.01) with the color yellow. Similarly, in Fig 21, two solutions are shown: The first one corresponds to the initial condition (*Y*, *Z*) = (1.60, 0.01) while the second one corresponds to the condition (*Y*, *Z*) = (1.70, 0.01) and the corresponding trajectories are in red and yellow respectively. In every circumstance to check the model sensitivity, we utilized *α*_1_, *α*_2_ is 0.5. In general, these solutions are super-nonlinear periodic wave solutions [42–44]. These results show that small variations in the given values greatly affect the final values, which proves that the model is sensitive to initial conditions. The non-linear parameter, specifically the Landau coefficient, has a high effect on the system.

## Chaotic pattern

In mathematics, a quasi-periodic wave is a type of motion generated by a dynamical system that comprises a restricted number of frequencies. The perturbation term *g*_0_ cos(*g*_1_*ξ*) is added to Eq (20) to make it more interesting. Thus, Eq (20) with the perturbation term can be expressed as [45]:

$$\begin{array}{c} \left\{ \begin{array}{cc} & \frac{dY}{d\xi }=Z,\\ & \frac{dZ}{d\xi }=\alpha_{1}Y-\alpha_{2}Y^{3}+g_{0}\;\text{cos}\left(g_{1}\xi \right),\\ & \alpha_{1}=\frac{z_{0}^{2}+2z_{3}z_{2}^{2}}{z_{2}^{4}}\;,\;\;\alpha_{2}=\frac{2\beta_{0}}{z_{2}^{2}}. \end{array}\right. \end{array}$$ (21)

**Fig 22:**
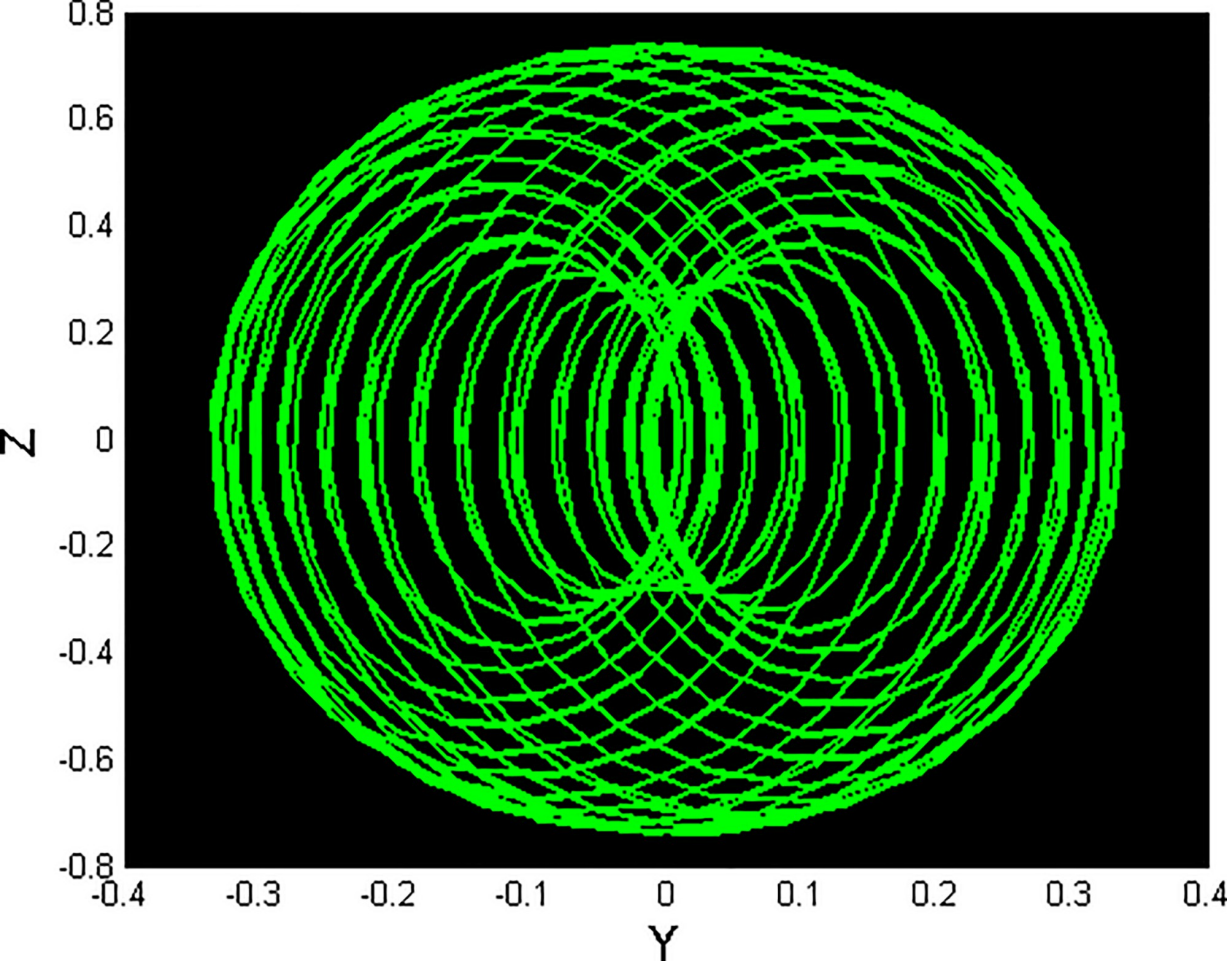
Chaotic phenomena with perturbed dynamical system (21) via 2D phase portrait using *g*_0_ = 1.20, and *g*_1_ = 3.

**Fig 23:**
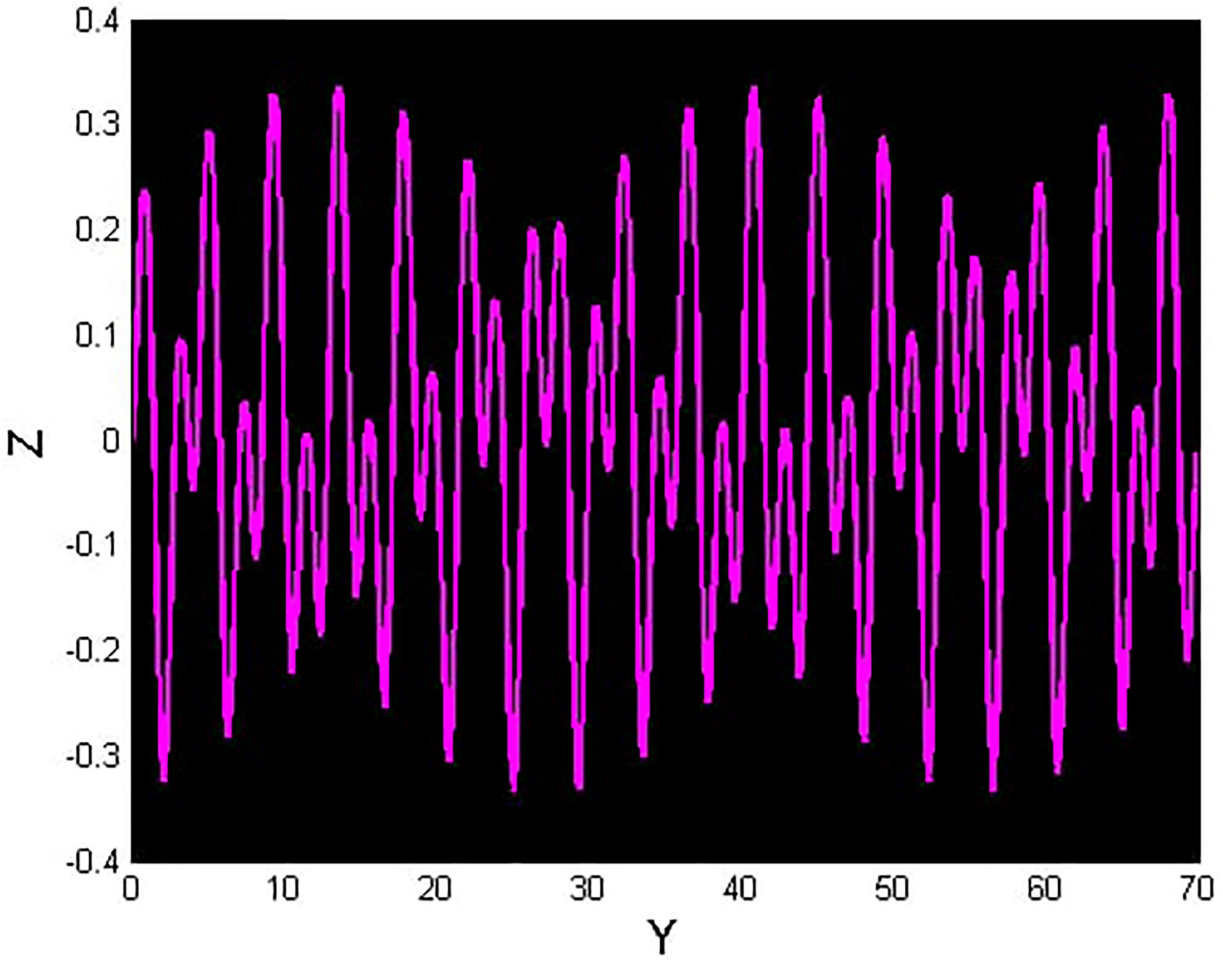
Chaotic phenomena with perturbed dynamical system (21) via time analysis using *g*_0_ = 1.20, and *g*_1_ = 3.

**Fig 24:**
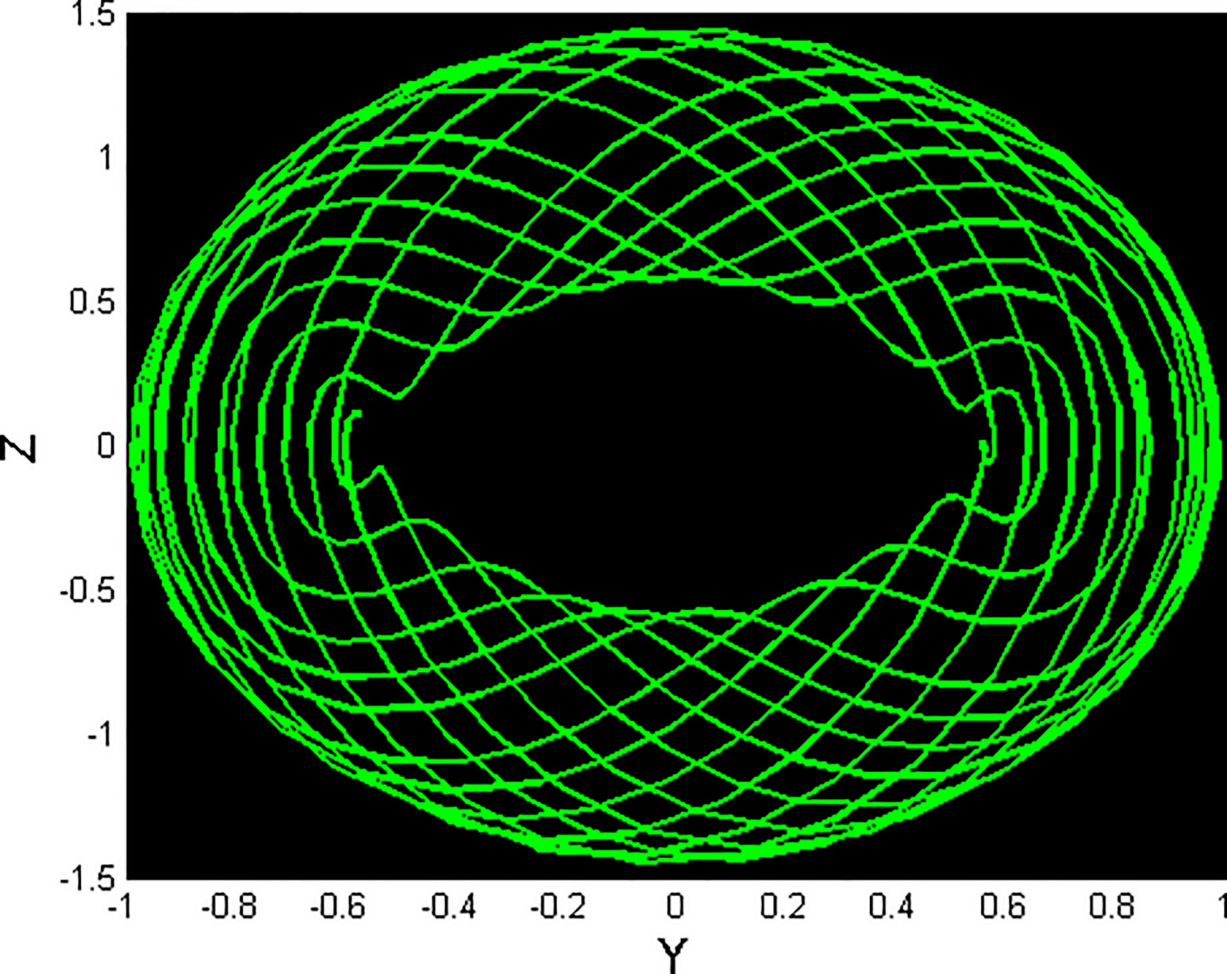
Chaotic phenomena with perturbed dynamical system (21) via 2D phase portrait using *g*_0_ = 1.20, and *g*_1_ = 2.

**Fig 25:**
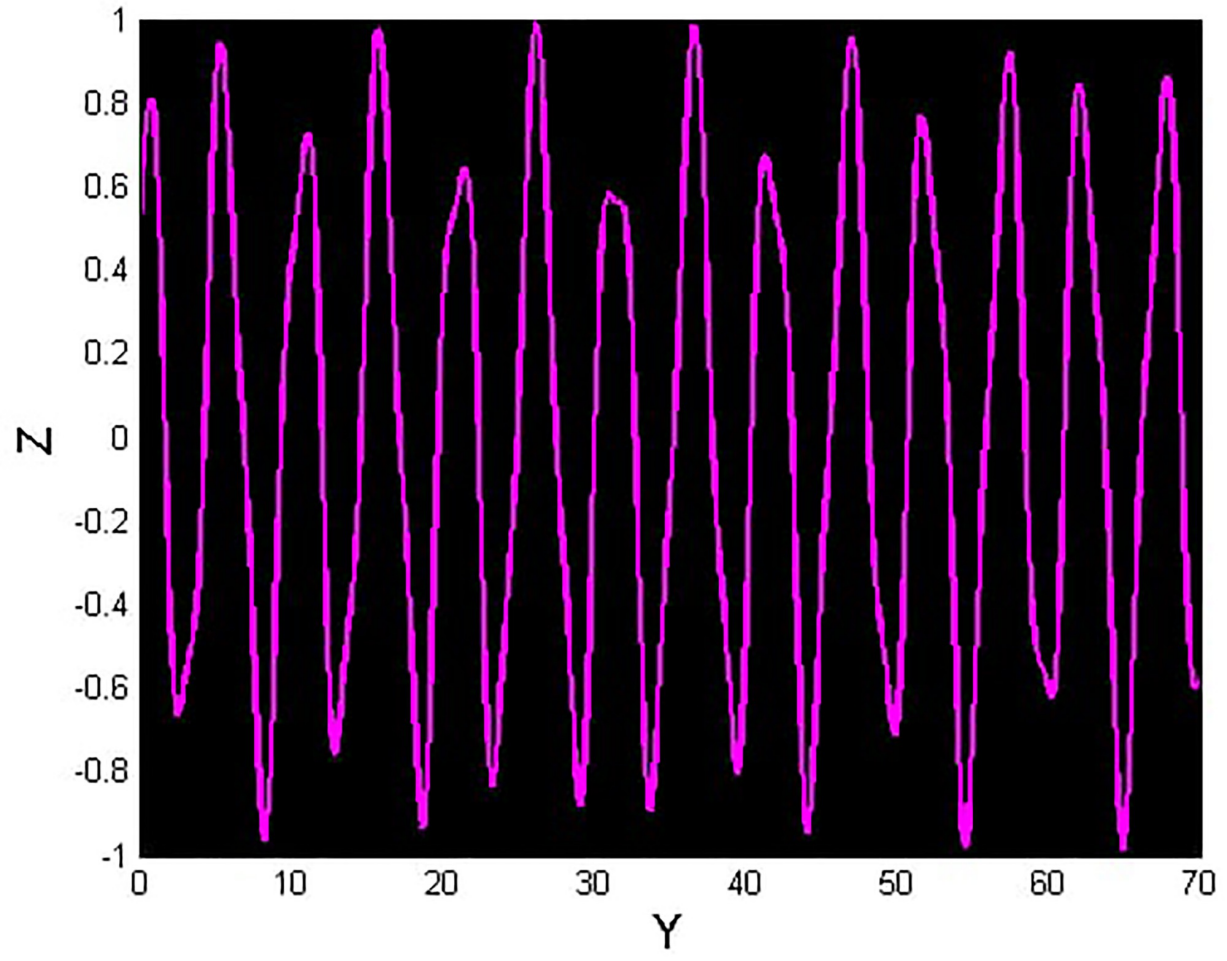
Chaotic phenomena with perturbed dynamical system (21) via time analysis using *g*_0_ = 1.20, and *g*_1_ = 2.

**Fig 26:**
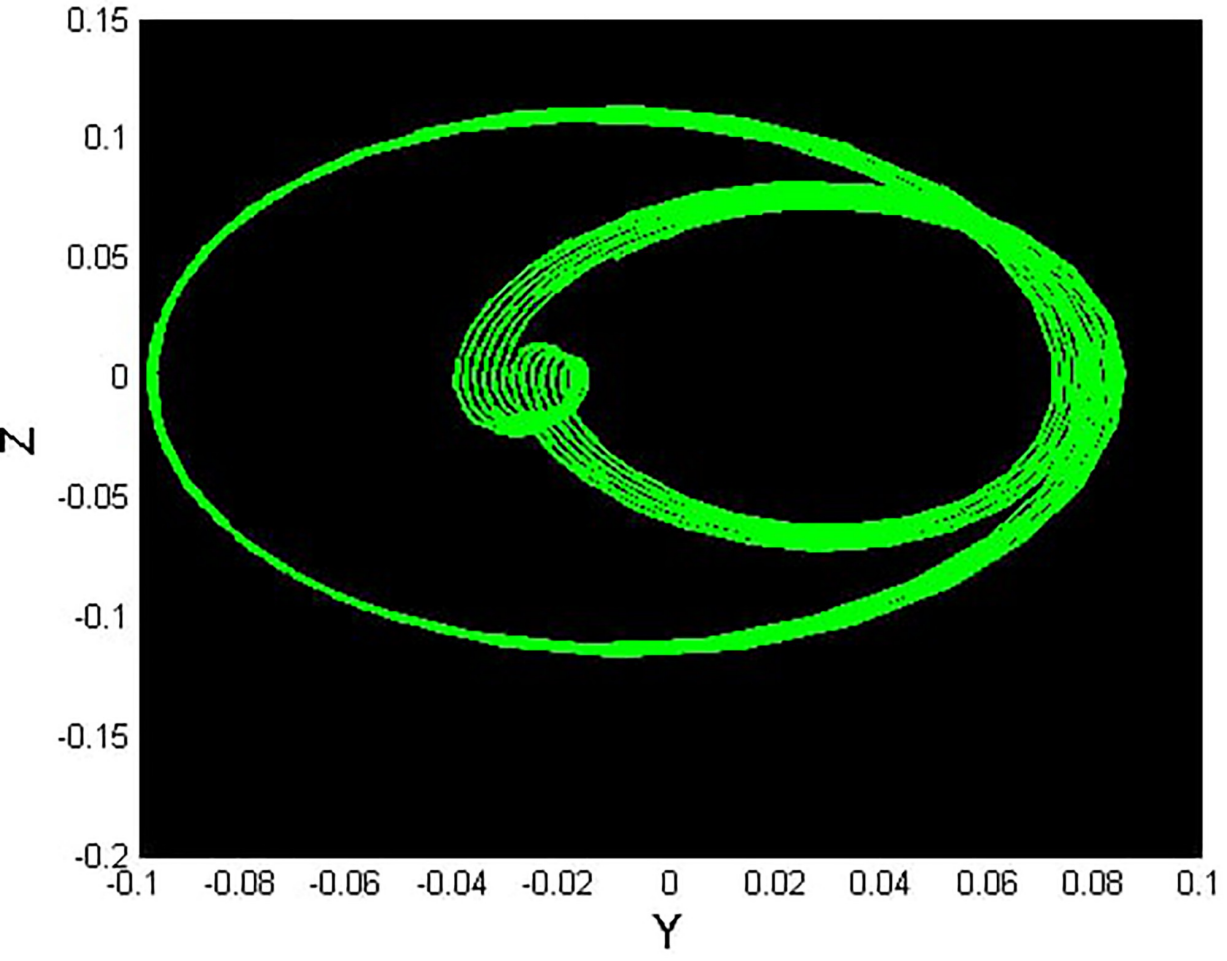
Chaotic phenomena with perturbed dynamical system (21) via 2D phase portrait using *g*_0_ = 0.05, and *g*_1_ = 1.

**Fig 27:**
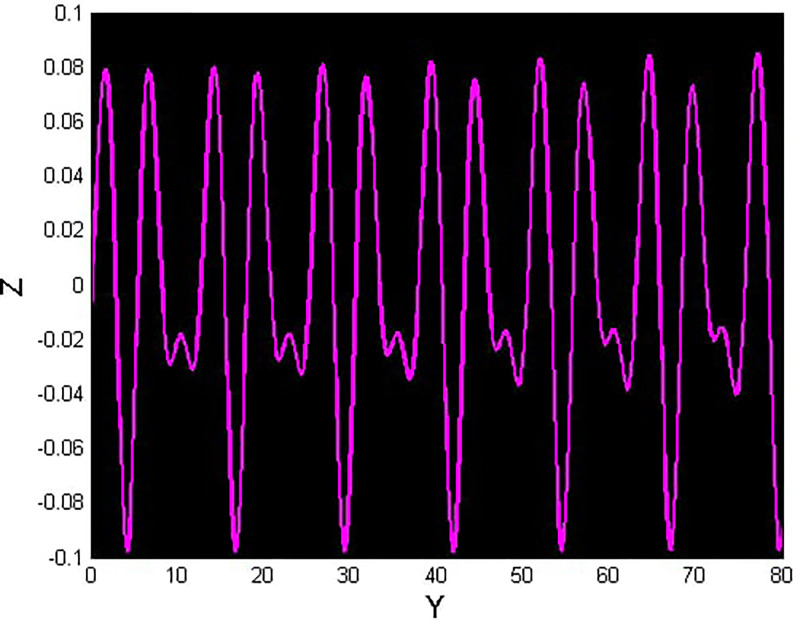
Chaotic phenomena with perturbed dynamical system (21) via time analysis using *g*_0_ = 0.05, and *g*_1_ = 1.

**Fig 28:**
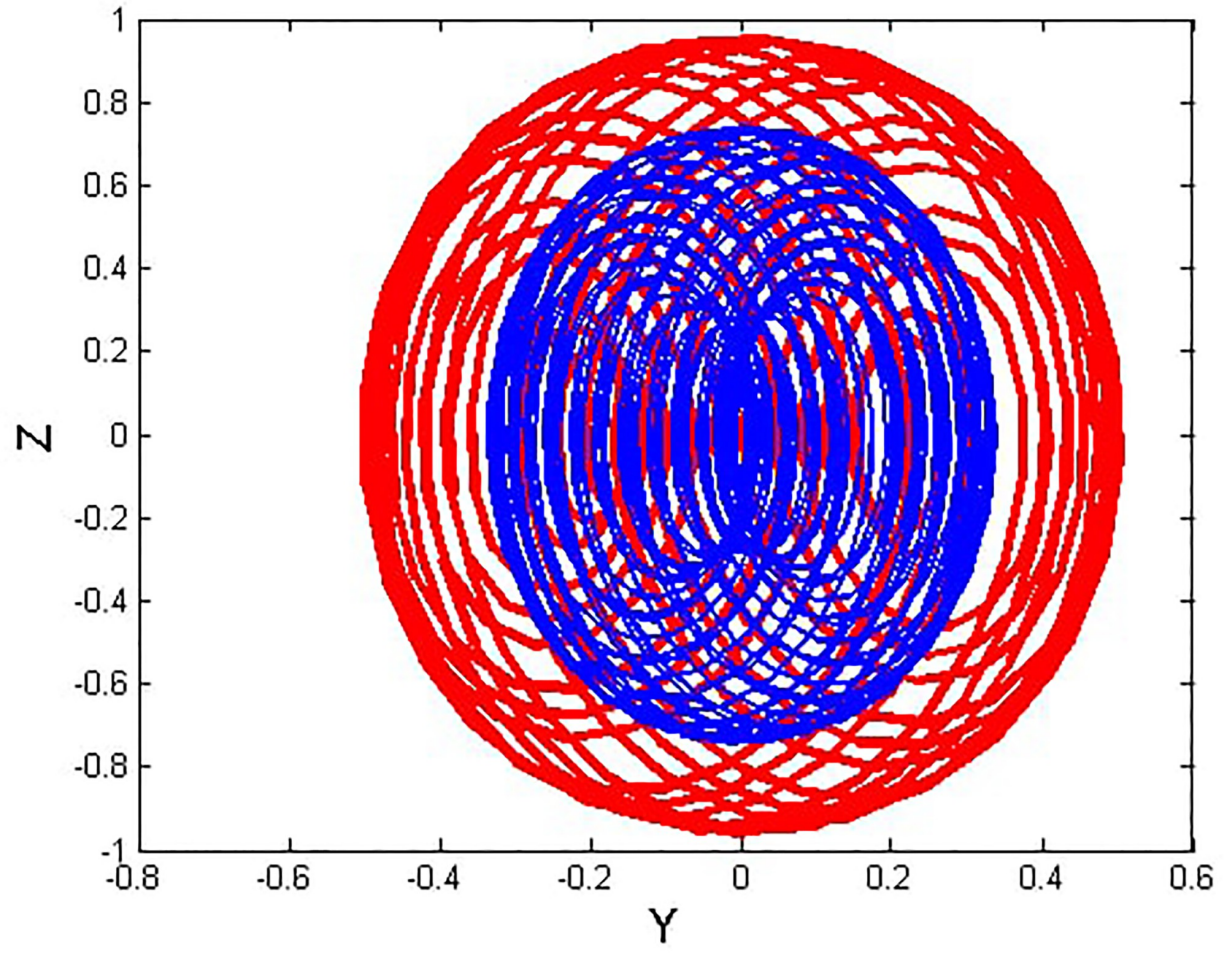
Chaotic phenomena with perturbed dynamical system (21) via multistability using (Y,Z) = (-0.01,0.05), (Y,Z) = (-0.02,0.05).

**Fig 29:**
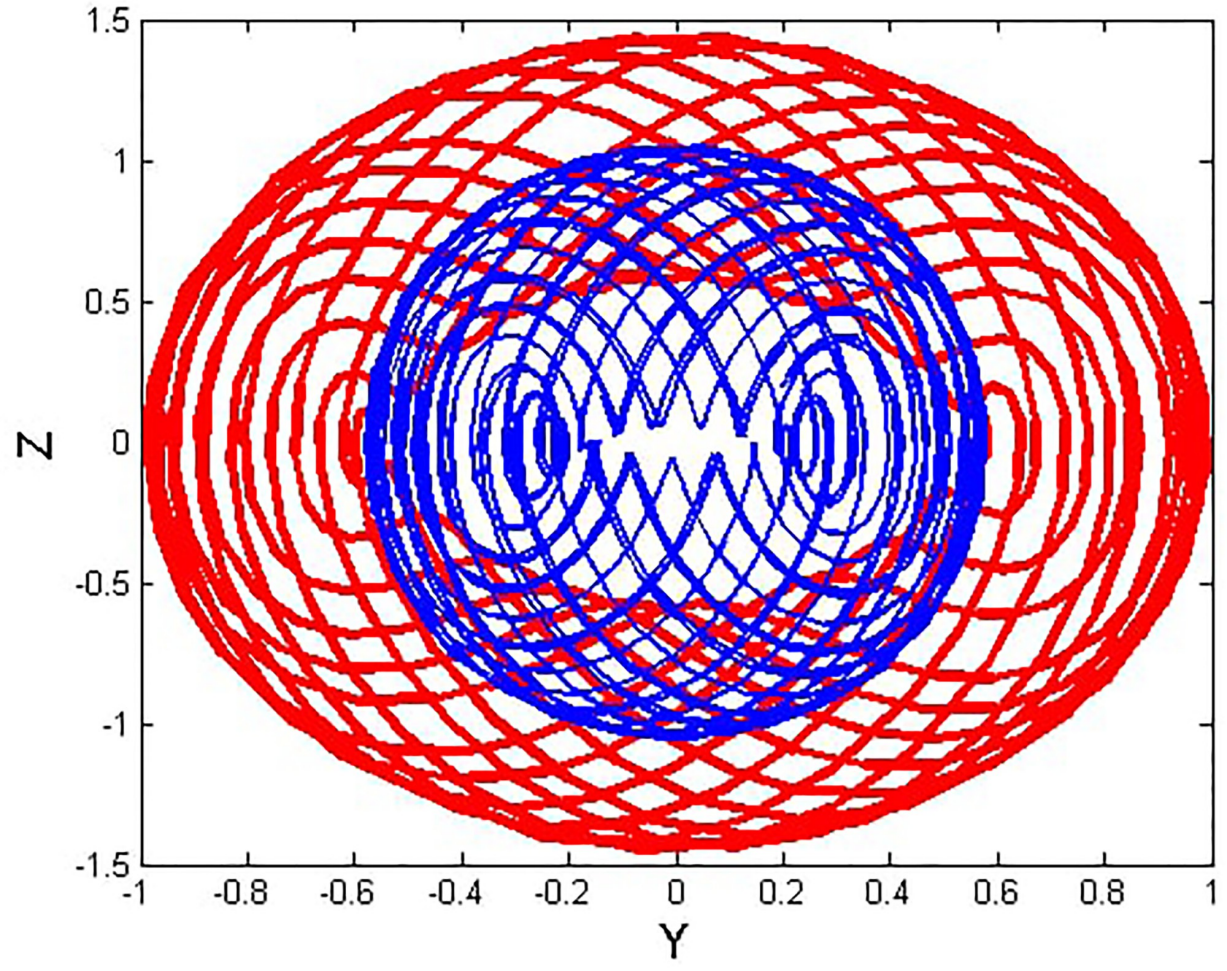
Chaotic phenomena with perturbed dynamical system (21) via multistability using (Y,Z) = (0.50,0.50), (Y,Z) = (0.20,0.20).

We will consider the external perturbation applied to the dynamical system (20) to be a function of *g*_0_ and *g*_1_. System (21) includes the trivial force, which is not involved in the system (20). The chaotic behavior of Eq (3) under perturbation by unknown variables will be investigated. We will maintain the visible limits of the system modifications and examine the impact of power and perturbation frequency on the IOPM. In Figs 22 and 23 shows a 2D phase portrait and a time series graph for *α*_1_ = −1.9, *α*_2_ = −1.0, *g*_0_ = 1.20, and *g*_1_ = 3. and initial conditions (*Y*, *Z*) = (−0.01, 0.05). The perturbed dynamical system (21) describes a chaotic pattern. In Figs 24 and 25 show a 2D phase portrait and a time series graph for *α*_1_ = −1.0, *α*_2_ = −1.0, *g*_0_ = 1.20, and *g*_1_ = 2. and initial conditions (*Y*, *Z*) = (0.50, 0.50). The perturbed dynamical system (21) exhibits chaotic pattern. A slight variation in initial values has no effect on the results, which supports the chaotic pattern. In Figs 26 and 27 show a 2D phase portrait and a time series graph for *α*_1_ = 1, *α*_2_ = −1.0, *g*_0_ = 0.50, and *g*_1_ = 1. and initial conditions (*Y*, *Z*) = (−0.01, 0.05). The perturbed dynamical system (21) exhibits chaotic pattern to assess the multistability of the system (21) by analyzing the phase portrait graph of the IOPM with the parametric values *α*_1_ = −1.9, *α*_2_ = −1.0, *g*_0_ = 1.20, and *g*_1_ = 3. We take initial conditions in this case are (*Y*, *Z*) = (−0.01, 0.05) in red and (*Y*, *Z*) = (−0.02, 0.05) in blue as shown in Fig 28. In Fig 29 show the multistability analysis under the parametric values *α*_1_ = −1.0, *α*_2_ = −1.0, *g*_0_ = −1.20, and *g*_1_ = 2. We take initial conditions in this case are (*Y*, *Z*) = (0.50, 0.50) in red and (*Y*, *Z*) = (0.60, 0.60) in blue. For all the observations, the IOPM has quasi-periodic behavior.

## Dynamical analysis

In this section, we confirm chaotic behavior using different tools such as bifurcation diagrams, Poincaré maps, Lyapunov exponents, and wave structures.

**Fig 30:**
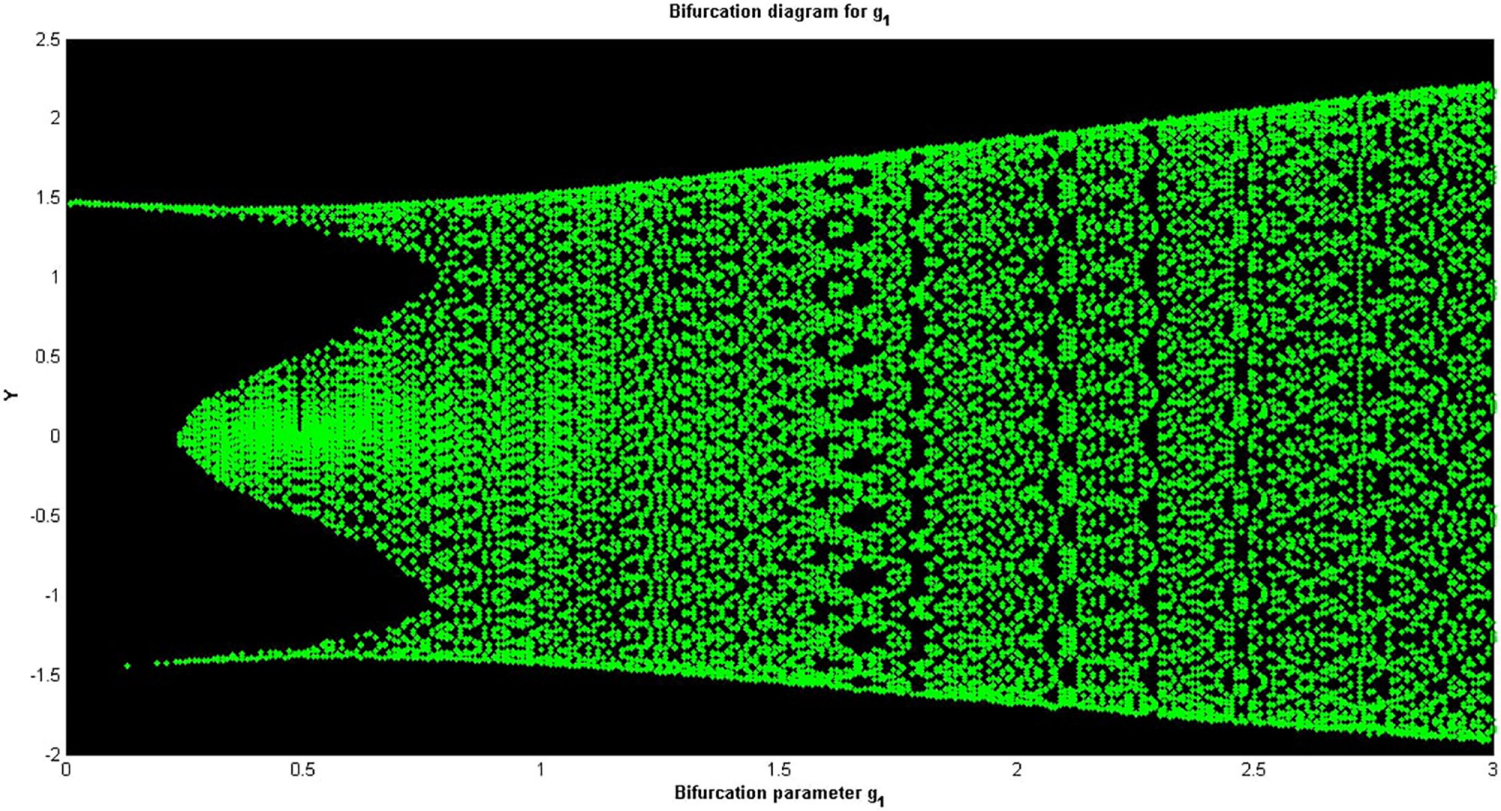
Analysing chaotic actions in system (21) using a bifurcation diagram between *Y* and *g*_2_ with physical quantities *g*_1_ = 0.5, *α*_1_ = 1, *α*_2_ = 1, and *α*_2_ under the starting scenario (0.2, 0.2).

**Fig 31:**
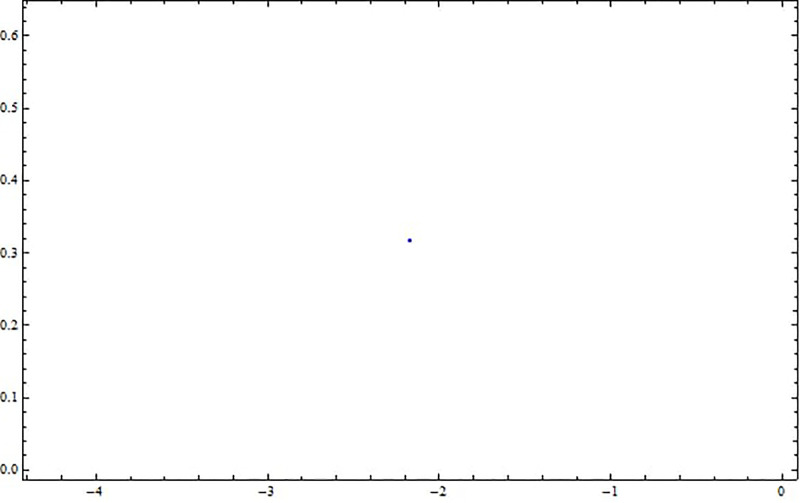
Poincaré map with characteristics *α*_1_ = 0.5, *α*_2_ = −0.5, and *g*_0_ = 0.5 for the nonlinear system (21) using $g_{1}=\frac{\pi }{6}$.

**Fig 32:**
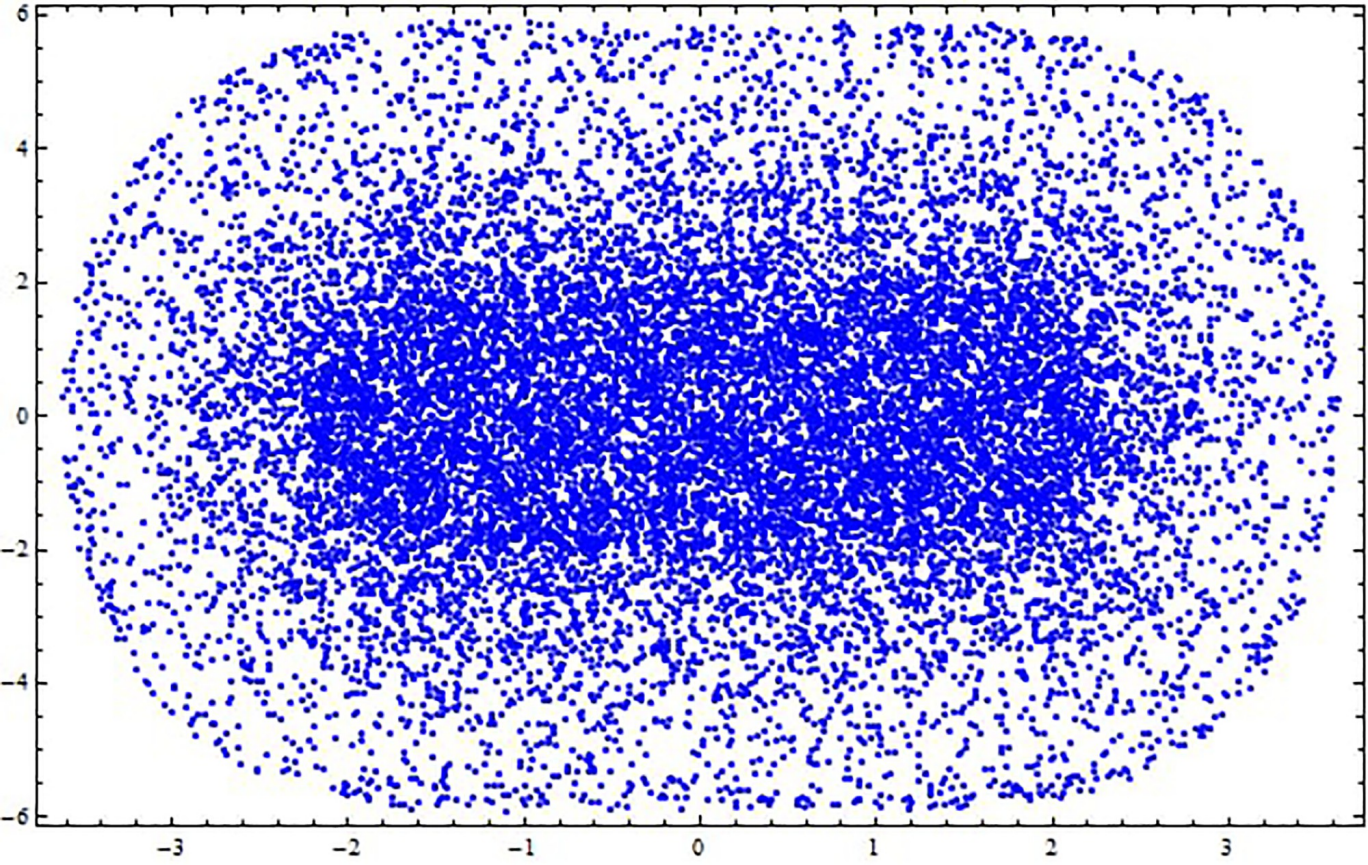
Poincaré map with characteristics *α*_1_ = 0.5, *α*_2_ = −0.5, and *g*_0_ = 0.5 for the nonlinear system (21) using $g_{1}=\frac{\pi }{2}$.

**Fig 33:**
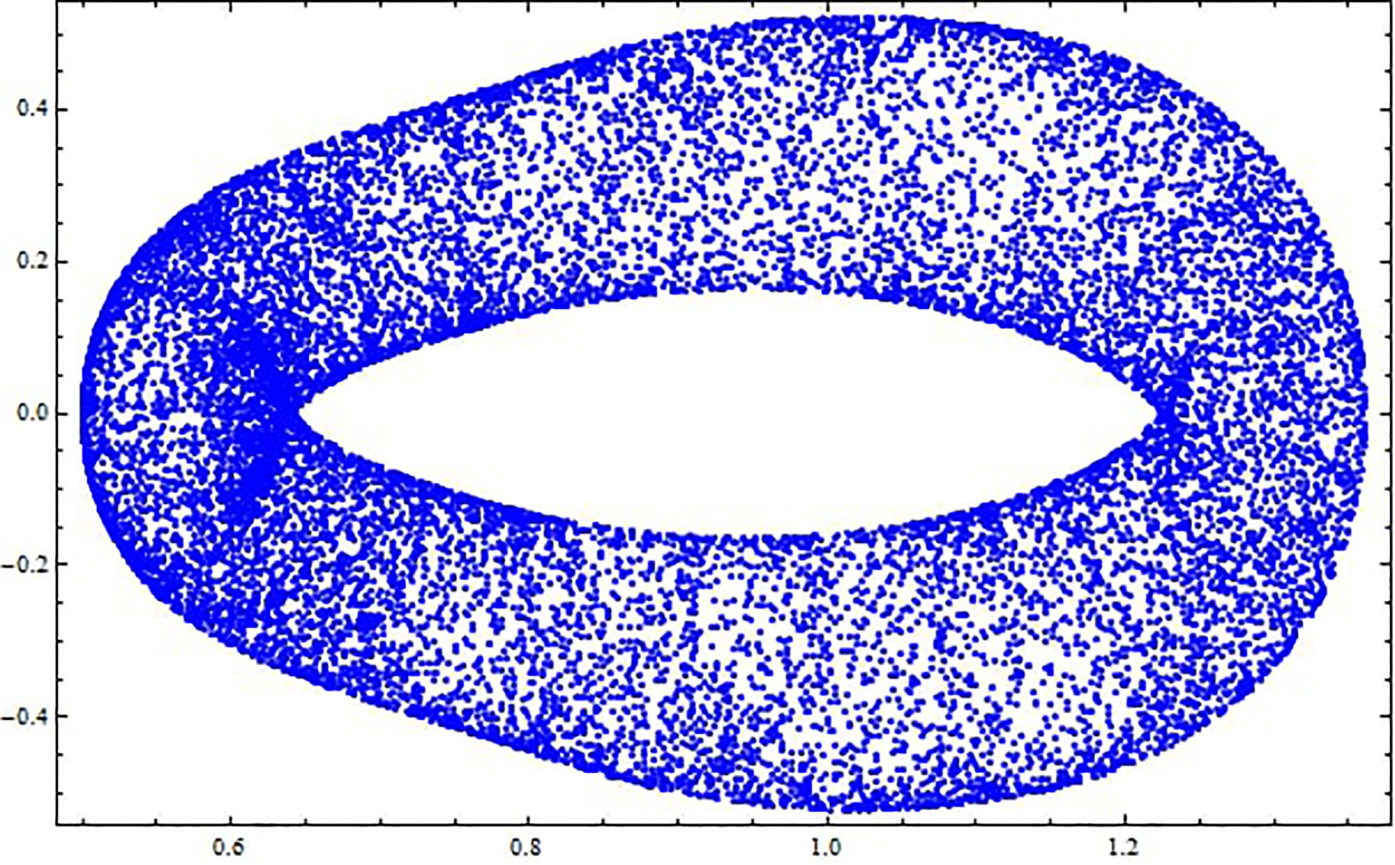
Poincaré map with characteristics *α*_1_ = 0.5, *α*_2_ = −0.5, and *g*_0_ = 0.5 for the nonlinear system (21) using *g*_1_ = *π*.

A bifurcation diagram is a graphical output that depicts the dynamical system and its behavior when changes occur to a parameter. For a perturbed dynamical system, the above parameter can be used to determine the strength or the level of perturbation. From the bifurcation diagram, one can determine various behaviors that the system may demonstrate, which include disequilibrium, in other words, chaos, limit cycles, or fixed points [46–48]. In Fig 30, *Y*–bifurcation diagrams of the perturbed dynamical systems (21) are shown by fixing *α*_1_ = 0.05, *α*_2_ = 0.05, *g*_1_ = 4.5, and *g*_0_ = [0, 3]. The high values of the non-linear parameter, such as the Landau coefficient, have a significant effect on the system. In this part, we have provided information on one of the methods of detecting chaos called the Poincaré map [49]. This technique involves converting an *m*^*th*^-order dynamical system into an (*m* − 1) order map that helps in analyzing stabilities and reducing the complexity of systems. It makes it possible to visualize various kinds of solutions such as periodic, chaotic, and quasi-periodic solutions as depicted in Figs 31–33 respectively. The Poincaré map has the following dynamic characteristics:

- A closed curve represents quasi-periodic solutions.
- Distinct points represent chaos solutions.
- Periodic solutions are represented by points.

Fig 31 shows periodic behavior with a stable, single point that forms a repeating pattern in the poincaré map, indicating predictable system dynamics. Fig 32 illustrates chaotic behavior with an irregular pattern, highlighting sensitivity to initial conditions, while Fig 33 displays quasi-periodic behavior, where trajectories form a complex, non-repeating structure.

The Lyapunov exponent is used to detect such chaos in a given dynamic system. Positive Lyapunov exponents in their case [50, 51] mean exponential divergence or chaos, and negative values mean convergence. Therefore, we have to evaluate the Lyapunov exponents for the given model of consideration. Thus, the Lyapunov exponents for the dynamic system (21) are determined as:

$$\mu_{1}=0.109511,\;\;\mu_{2}=-0.109511.$$

**Fig 34:**
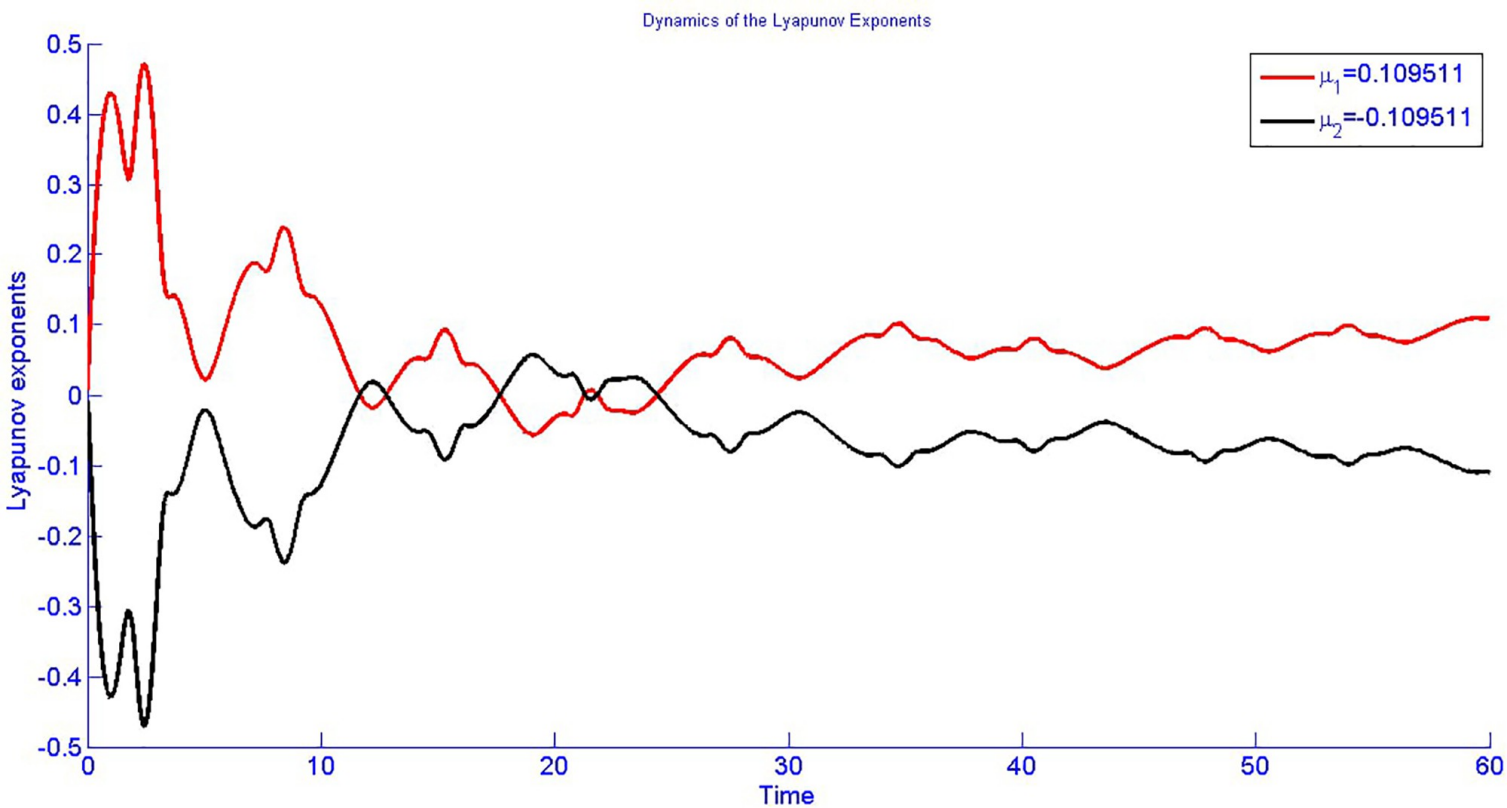
Analysing chaotic actions in system (21) using a Lyapunov exponents between *Y* and *g*_2_ with physical quantities *g*_0_ = 0.05, *g*_1_ = 0.5, *α*_1_ = 1, *α*_2_ = 1, and *α*_2_ under the starting scenario (0.2, 0.2).

**Fig 35:**
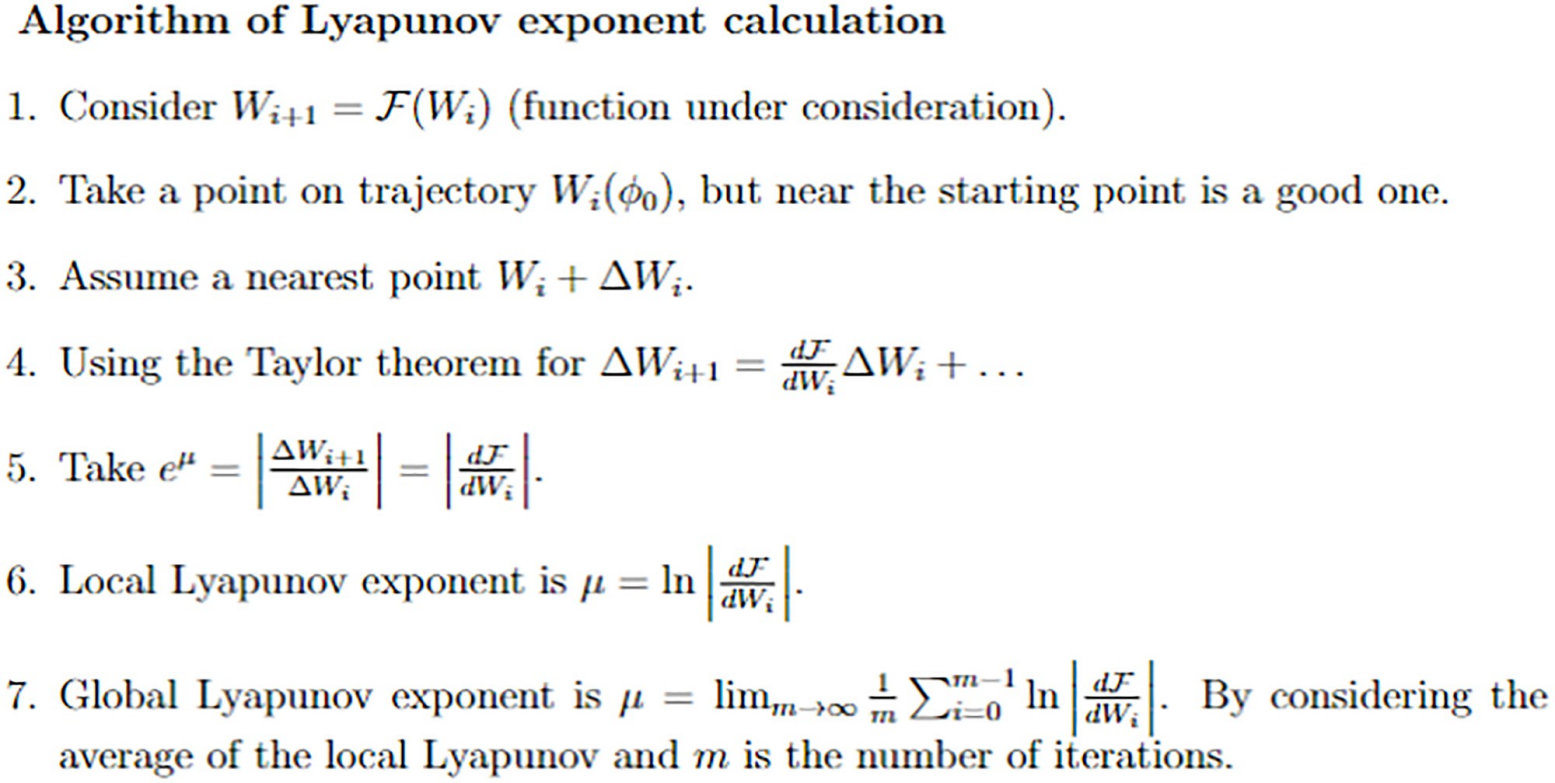
Overview of the algorithm used to calculate Lyapunov exponents for assessing the stability and chaotic behavior of dynamical systems.

For a better understanding of the complexity of the system (21) and its tendency to oscillate chaotically, the calculated Lyapunov exponents are shown in Fig 34 depending on time. From the analysis of the considered model, illustrated in Fig 34, the exponent is positive, hence, pointing to the chaotic nature of the model. Furthermore, a brief description of the algorithm for calculating Lyapunov exponents is presented in Fig 35.

**Fig 36:**
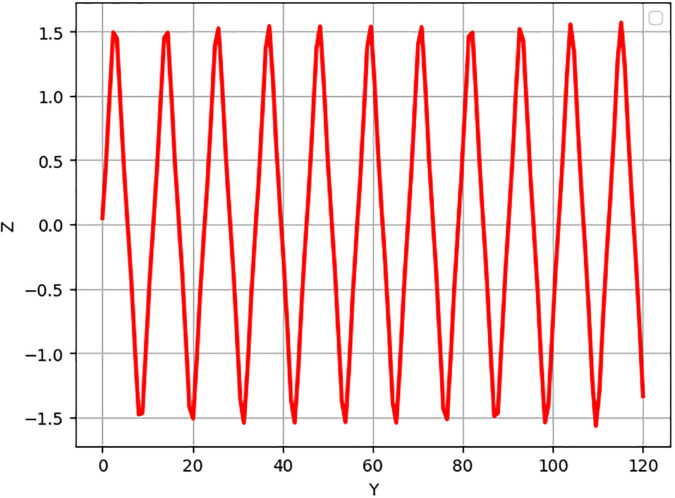
Non-linear periodic wave structure of (20) for *α*_1_ = 1, and *α*_2_ = 1, with Initial condition (0.50, 0.1).

**Fig 37:**
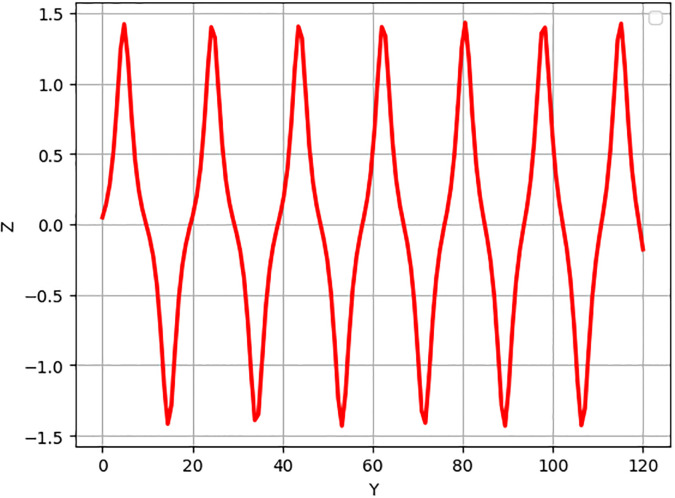
Super nonlinear periodic structure of (20) for *α*_1_ = 1, and *α*_2_ = 1, with Initial condition (0.05,0.5).

Non-linear wave solutions of systems (20) can be investigated using phase plane analysis depending on the physical parameters *α*_1_ = 1 and *α*_2_ = 1, which results in different phase plots with multiple fixed points and their stability [52, 53]. We can find trajectories in these phase plots are associated with wave solutions, with the abbreviation of NPO for non-linear periodic orbit, NHO for non-linear homoclinic orbit, SNHO for super-nonlinear homoclinic orbit SNPO, and super-nonlinear periodic orbit. These trajectories are in direct relationship with wave solutions and, therefore, imply the necessity to find all possible super-nonlinear trajectories of the given system (20) with the help of variation of suitable physical parameters. Figs 36 and 37 present the system known as super-nonlinear periodic wave solutions in Fig 36 and non-linear periodic waves in Fig 37 which shows that the waves can exhibit a lot of behaviors.

## Results as well as discussions

The IOPM effectively describes non-linear optical phenomena, including optical solitons and chaos. The model’s application provides insights into quasi-periodic and chaotic patterns in non-linear optics.

- Quasi-Periodic Patterns: The IOPM model analyzes quasi-periodic patterns, which exhibit regularity without exact periodicity. Understanding these patterns aids in designing and controlling optical devices and systems, enhancing their performance.
- Chaotic Patterns: The IOPM model captures chaotic behavior in optical systems arising from non-linear light wave interactions and dispersion.
- Summary Studying quasi-periodic and chaotic patterns using the IOPM model enhances our understanding of optical systems, informing the design and optimization of optical devices and communication technologies.

## Conclusion

This study offers an in-depth exploration of the IOPM, focusing on its dynamic behavior through various mathematical and analytical techniques. As an extension of the Black-Scholes model, the IOPM incorporates non-linear Schrödinger equations to model controlled Brownian motion, which introduces complexities such as soliton solutions, chaotic behavior, and sensitivity to initial conditions. The motivation for this research stems from the need to better understand and predict the behavior of nonlinear systems in various applications, ranging from engineering to financial models and fiber optic communication systems. By transforming the partial differential equation into an ordinary differential equation, we applied the $\left(\frac{G^{\prime }}{G}\right)$ method to successfully derive exact soliton solutions. These solutions, including singular-kink, periodic, hyperbolic, trigonometric, exponential, and complex forms, are of great importance for understanding the behavior of wave structures in nonlinear systems. Soliton solutions, in particular, play a vital role in fields such as fluid dynamics, optical fibers, and plasma physics, where the preservation of wave shapes over long distances is crucial. In Figs 1–18 visually demonstrate these soliton solutions, providing valuable insights into the wave dynamics of the IOPM.

Additionally, the introduction of an external periodic force into the dynamical system allowed us to detect and analyze chaotic and quasi-periodic behavior using several tools. Poincaré maps, time series, and multistability analyses were pivotal in revealing the complex dynamics of the system. The Poincaré map, in particular, is a powerful tool in dynamical systems analysis, offering a discrete representation of a system’s trajectory by plotting intersections with a lower-dimensional subspace. This technique is crucial for identifying periodic, quasi-periodic, and chaotic behavior, providing a clearer understanding of the system’s long-term dynamics. In this study, the Poincaré maps effectively revealed the presence of periodic, quasi-periodic, and chaotic patterns, offering a visual representation of how the system transitions between different types of behavior. Time series analysis further enhanced our understanding of the system’s evolution over time, highlighting the transition from regular to chaotic behavior. Time series plots are critical in many applications, including control systems, climate modeling, and financial markets, where predicting future behavior based on past patterns is essential. Multistability analysis, another key tool used in this study, uncovered regions where the system exhibited multiple stable states, underscoring the complex nature of nonlinear systems and their susceptibility to initial conditions. A key finding is the system’s sensitivity to initial conditions, where small parameter changes yield vastly different outcomes, indicating chaotic behavior. This sensitivity is crucial for understanding the unpredictability of nonlinear systems, especially in applications such as secure communications, where chaotic signals can be exploited for encryption, or in engineering systems where stability is critical. The chaotic behavior identified in this study through phase plots, Poincaré maps, and time series emphasizes the unpredictable and highly sensitive nature of the IOPM. Chaotic dynamics, often seen as a disadvantage due to their unpredictability, can also be harnessed for practical applications, such as secure communication systems, where chaotic signals can ensure data security by making unauthorized decoding exceedingly difficult. Furthermore, chaotic behavior has applications in biological systems, weather prediction, and market dynamics, where understanding and managing sensitivity and unpredictability are vital. In addition to chaotic dynamics, the Landau coefficient was found to have a significant impact on the system. The Landau coefficient influences the stability of solutions in nonlinear systems, and its role in this study highlighted its ability to control the transition between regular and chaotic states. Understanding the effect of the Landau coefficient can be particularly useful in designing systems that need to transition smoothly between different dynamic behaviors or maintain stability under varying conditions.

Finally, the application of the Runge-Kutta method to solve the derived ordinary differential equations enabled us to capture the super-nonlinear and nonlinear periodic wave structures of the system. The ability to model such complex wave dynamics has practical importance in numerous fields, including fluid mechanics, optical fiber technology, and plasma physics, where nonlinear wave solutions are used to describe phenomena such as shock waves, turbulence, and energy transmission.

## Future task

Studying this model becomes more complex at higher non-linearity parameter values. Our next goal is to explore bifurcation and chaos theory to understand these complexities. Researchers aim to use various analytical methods to uncover dynamic characteristics, opening up exciting avenues for future exploration. Researchers can work on conservation laws, Lie and stability analysis, lump solutions, etc.
